# PDGFRα plays a crucial role in connective tissue remodeling

**DOI:** 10.1038/srep17948

**Published:** 2015-12-07

**Authors:** Shinjiro Horikawa, Yoko Ishii, Takeru Hamashima, Seiji Yamamoto, Hisashi Mori, Toshihiko Fujimori, Jie Shen, Ran Inoue, Hirofumi Nishizono, Hiroshi Itoh, Masataka Majima, David Abraham, Toshio Miyawaki, Masakiyo Sasahara

**Affiliations:** 1 Department of Pathology, Graduate School of Medicine and Pharmaceutical Sciences, University of Toyama, Toyama 930-0194, Japan; 2 Department of Pediatrics, Graduate School of Medicine and Pharmaceutical Sciences, University of Toyama, Toyama 930-0194, Japan; 3 Department of Molecular Neuroscience, Graduate School of Medicine and Pharmaceutical Sciences, University of Toyama, Toyama 930-0194, Japan; 4 Division of Embryology, National Institute for Basic Biology, Okazaki 444-8787, Japan; 5 Division of Animal Experimental Laboratory, Life Science Research Center, University of Toyama, Toyama 930-0194, Japan; 6 Department of Molecular Pathology, Graduate School of Medicine, Yamaguchi University, Ube 755-8505, Japan; 7 Department of Pharmacology, School of Medicine, Kitasato University, Kanagawa 252-0374, Japan; 8 Division of Medicine, University College London, Royal Free Campus, London, NW3 2PF, UK

## Abstract

Platelet derived growth factor (PDGF) plays a pivotal role in the remodeling of connective tissues. Emerging data indicate the distinctive role of PDGF receptor-α (PDGFRα) in this process. In the present study, the *Pdgfra* gene was systemically inactivated in adult mouse (α-KO mouse), and the role of PDGFRα was examined in the subcutaneously implanted sponge matrices. PDGFRα expressed in the fibroblasts of *Pdgfra*-preserving control mice (Flox mice), was significantly reduced in the sponges in α-KO mice. Neovascularized areas were largely suppressed in the α-KO mice than in the Flox mice, whereas the other parameters related to the blood vessels and endothelial cells were similar. The deposition of collagen and fibronectin and the expression of *collagen 1a1* and *3a1* genes were significantly reduced in α-KO mice. There was a significantly decrease in the number and dividing fibroblasts in the α-KO mice, and those of macrophages were similar between the two genotypes. *Hepatocyte growth factor* (*Hgf*) gene expression was suppressed in *Pdgfra*-inactivated fibroblasts and connective tissue. The findings implicate the role of PDGFRα-dependent ECM and HGF production in fibroblasts that promotes the remodeling of connective tissue and suggest that PDGFRα may be a relevant target to regulate connective tissue remodeling.

Connective tissue remodeling (CTR) is an essential process during the healing of wounds and fibrotic diseases as well as in the progression of tumors^1^. The process of remodeling is affected by numerous factors, including age, nutritional deficiencies, metabolic diseases, the type of tumor, and others^2^,^3^,^4^. Sequences of cellular events are involved in CTR, such as the recruitment of inflammatory cells, fibroplasia, new blood vessel formation, and deposition of extra-cellular matrix (ECM). Further understanding of the underlying molecular mechanisms of CTR is essential for the care of impaired healing processes such as in case of intractable ulcers as well as for the development of anti-fibrotic therapies and anti-cancer strategies.

CTR is regulated by a number of growth factors and cytokines^5^. Among the regulators are members of the PDGF family, including PDGF-A, -B, -C and -D. These PDGF-ligands are assembled as disulfide-linked homo- or hetero-dimers, and they bind to and activate two types of PDGF receptors, PDGFRα and PDGFRβ^6^. The PDGFs have been assumed to carry out crucial functions in several stages of the wound healing process^7^. The role of the PDGF-B/PDGFRβ interaction in promoting the recruitment of fibroblasts, pericytes, and endothelial cells to enhance wound healing in experimentally induced rodent skin injury has been well-documented^8^,^9^,^10^. On similar lines, recombinant human variants of PDGF-BB (also known as, Becaplermin) are FDA-approved drugs that have been successfully applied against intractable ulcers^11^.

On the other hand, emerging data are indicating the relevance of PDGFRα in CTR^12^. Enhancement of the intrinsic PDGFR signal, evoked by mutational activation of *Pdgfra,* but not *Pdgfrb*, systemically induced fibroblastic hyperplasia and increased ECM deposition similar to that observed in collagen diseases^13^. A good correlation has been observed between the upregulation of the PDGF-A/PDGFRα axis and enhanced healing of skin wounds observed in mice in which the gene for the nuclear factor I-C has been knocked out^14^. Additionally, PDGFRα binds to PDGF-C with high affinity and has been suggested to mediate angiogenesis and fibroblastic cell proliferation in ischemic tissue and tumor stroma^2^,^15^,^16^,^17^,^18^. Thus, it has been suggested that PDGFRα might contribute to CTR through a different mechanism than that of PDGFRβ. However, the functional relevance of PDGFRα in CTR remains largely unknown and demands further exploration.

In the present study, in order to understand the role of PDGFRα in CTR, we established a mouse line (α-KO) in which the *Pdgfra* gene was ubiquitously inactivated by tamoxifen-induced Cre recombinase. Then, the effect of the knockdown was examined in CTR using the sponge-implant model, which model has been adopted for the accurate quantification of angiogenic and fibrogenic responses *in vivo*^19^. While PDGFRα expression was principally observed in fibroblasts within the ingrowing connective tissues of the sponge matrices implanted in the control mice (Flox mice), it was effectively depleted in the fibroblasts of the α-KO mice. Decreased angiogenesis and ECM deposition were the most striking phenotypes during CTR of the α-KO mice. These phenotypes were different from those obtained after PDGFRβ inhibition^8^,^10^ and suggest a distinctive role for PDGFRα during CTR.

## Results

### Tamoxifen suppresses PDGFRα expression in skin fibroblasts of *Pdgfra*-inactivated (α-KO) mice

Neither male nor female mice, with tamoxifen-induced systemic inactivation of *Pdgfra,* showed apparent adverse effects during experiments. The body weights were similar between the two mouse genotypes within the same sex (Supplementary Fig. 1a). In normal skin of the Flox mice, even after tamoxifen treatment, PDGFRα was expressed in the spindle-shaped fibroblasts of the dermis and the subcutaneous connective tissue (Supplementary Fig. 1b, left). In contrast, PDGFRα expression was largely suppressed in the α-KO mice (Supplementary Fig. 1b, right).

### Tamoxifen suppresses PDGFRα expression in ingrowing connective tissue of α-KO mice

The role of PDGFRα in CTR was examined by the use of sponge implantation model in mice. Because tamoxifen has an estrogen-like activity, both sexes of mice were subjected to the initial part of the study. As the ingrowth of connective tissue was very small at day 7 (data not shown), tissue responses were analyzed at days 14 and 28 after implantation. Sponge implanted in the back skin was excised after 14 days (Supplementary Fig. 2a, upper), and the H&E staining of the cut surface indicated abundant ingrowth of the newly synthesized connective tissue, which consisted of a mixture of round-shaped inflammatory cells, spindle-shaped cells and blood vessels (Supplementary Fig. 2a, lower).

The efficiency of gene inactivation was examined in the implanted sponge matrices. The level of *Pdgfra* mRNA was largely decreased in the α-KO mice, compared with that in the Flox mice at days 14 and 28 in both sexes, and the differences between the two genotypes were significant in real-time PCR analyses (Supplementary Fig. 2b). The level of *Pdgfrb* mRNA was significantly lower in α-KO than in Flox mice at day 14 in both sexes, and at day 28 in males, but was similar between two genotypes at day 28 in females (Supplementary Fig. 2b). The level of PDGFRα protein in sponge was largely suppressed in α-KO mice, compared with that in the Flox mice at days 14 and 28 in both sexes, and the differences between two genotypes were found to be significant in western blotting (Supplementary Fig. 2c). The level of PDGFRβ protein was significantly lower in the α-KO mice than in the Flox mice in males, but was similar in both genotypes in females at days 14 and 28 (Supplementary Fig. 2d).

### Expression of PDGFRα was suppressed in the fibroblast of α-KO mice

**Figure 1:**
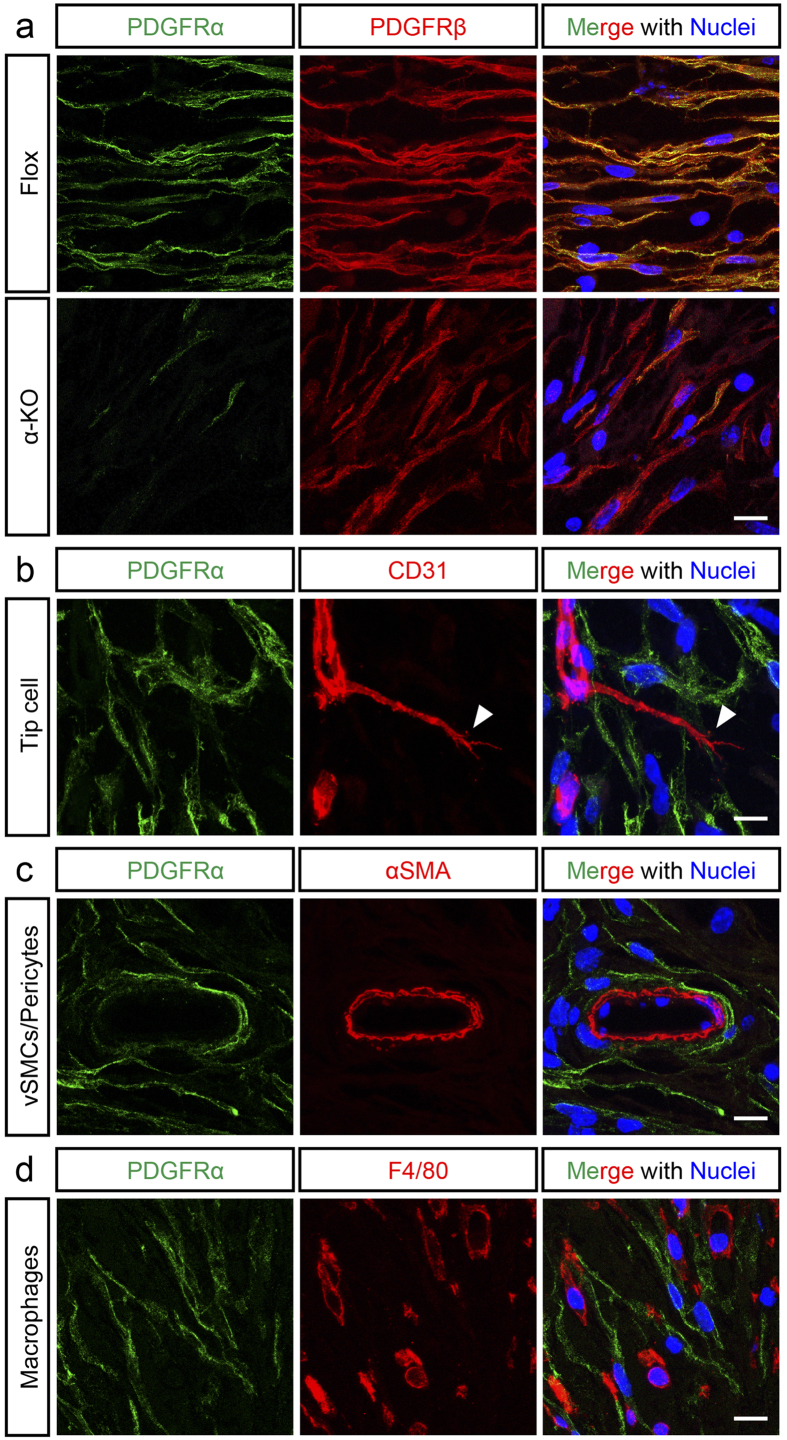
Immunofluorescence analysis of PDGFRα expression in implanted sponge. (**a**) Immunofluorescence of PDGFRα (green) and PDGFRβ (red) in Flox mice (upper row) and α-KO mice (bottom row) with nuclear staining (Hoechst, blue). (**b**–**d**) Multicolor immunofluorescence analysis of PDGFRα expression in Flox mice. Co-staining of PDGFRα (green) and CD31 (red; **b**), PDGFRα (green) and αSMA (red; **c**), PDGFRα (green) and F4/80 (red, d). The nuclei were stained with Hoechst (blue, **b**–**d**). PDGFRα was not expressed in endothelial cells, including specific tip cells, vascular smooth muscle cells/pericytes, and macrophages, all of which are major components of ingrowing connective tissue together with fibroblasts. Scale bars indicate 10 μm, respectively.

Immunofluorescence of the ingrowing connective tissue within sponge matrices from the Flox mice detected PDGFRα within spindle-shaped fibroblast-like cells, and it was colocalized with PDGFRβ in these cells (Fig. 1a, upper row). PDGFRα immuno-positivities were largely decreased, leaving PDGFRβ staining positive in these spindle-shaped cells in the α-KO mice on the 14th day after implantation (Fig. 1a, bottom row). In the double immunofluorescence with cell-type specific marker, PDGFRα was not colocalized with CD31, a marker of vascular endothelial cells in the Flox mice (Fig. 1b). Notably, PDGFRα was not detected in CD31-positive-mimicking tip cells, a subset of vascular endothelial cells (arrowhead in Fig. 1b)^20^. Similarly, PDGFRα was not detected in the blood vessel-associated alpha smooth muscle actin (αSMA)-positive cells that correspond to the vascular smooth muscle cells and/or pericytes (Fig. 1c). PDGFRα did not also colocalize with F4/80, a macrophage-specific marker (Fig. 1d). The results suggest that PDGFRα was mainly expressed in spindle-shaped fibroblasts in the Flox mice, as had been reported previously^8^,^10^, but was effectively suppressed in the newly synthesized connective tissues within sponge matrices of the α-KO mice.

### Reduced angiogenesis in α-KO mice

**Figure 2:**
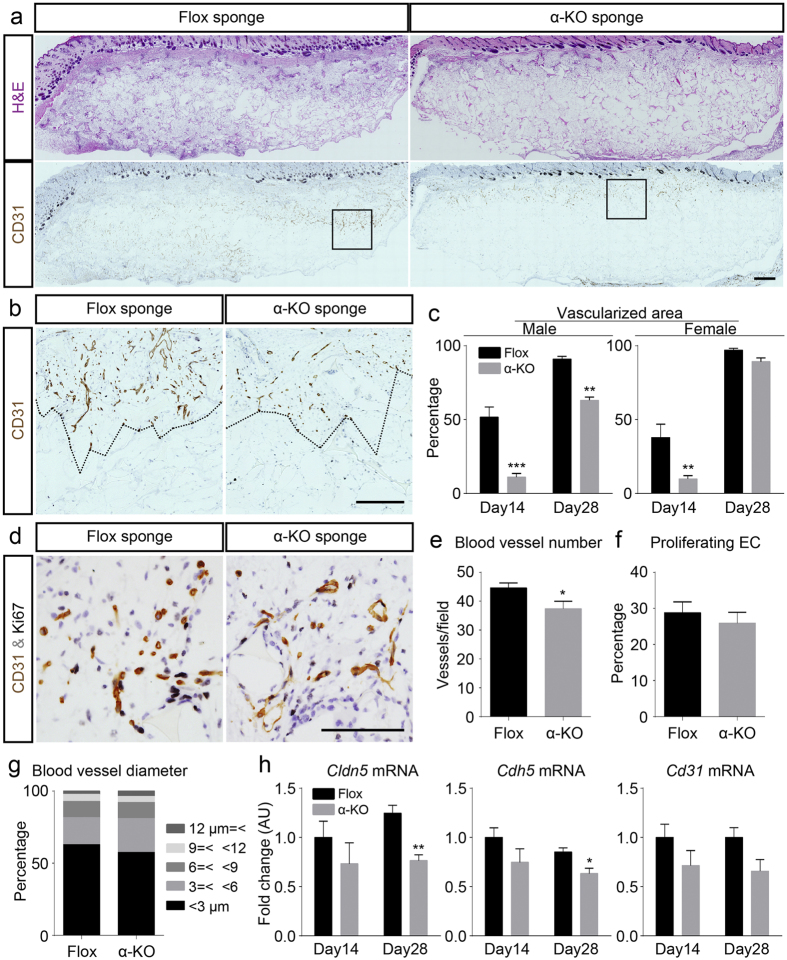
Angiogenesis is suppressed in implanted sponges in α-KO mice. (**a**–**c**) Immunohistochemical analysis of blood vessel formation in implanted sponge. (**a**) H&E-stained cross-sectional area of implanted sponges at day 14 after implantation (upper row) and immunohistochemistry of CD31 (brown) in the adjacent sections (bottom row). Boxed areas in (**a**) show as high magnification panels in (**b**) with the vascularized areas delineated by dotted lines. Using Flox mice as the control (**a**, left), CD31 positive vascularized areas in α-KO (**a**, right) were examined in male (**c**, left) and female (**c**, right) on days 14 and 28 after implantation. n = 4–5 mice per group. (**d**) Double immunostaining of CD31 (brown) and Ki67 (black) and counterstaining with hematoxylin (pale blue) in ingrowing connective tissue. The density of CD31-positive blood vessels is higher in the Flox (left) as compared with that in the α-KO (right) mice. (**e**–**g**) Morphometric analysis of angiogenesis and endothelial cell proliferation in connective tissues. Number of blood vessels in vascularized areas (**e**), Ki67 positivity in CD31-positive endothelial cells (**f**), and cumulative percentages of blood vessel diameters (**g**) of both genotypes; n = 4 per group. (**h**) Real-time PCR analyses of mRNA expression levels of endothelial cell-related gene *Cldn5*, *Cdh5*, and *Cd31* in sponges of Flox and α-KO mice. Male mice were analyzed in (**d**–**h**). *p < 0.05 versus Flox mice; **p < 0.01 versus Flox mice; ***p < 0.001 versus Flox mice. Scale bars indicate 1 mm (**a**), 500 μm (**b**), and 100 μm (**d**).

Newly synthesized connective tissue that had grown into the implanted sponge matrices was histologically compared between the two mouse genotypes. The growth-end of CD31-positive blood vessels was traced in CD31 immuno-stained sections, and the vascularized areas within the sponge matrices were calculated (Fig. 2a,b). The CD31-positive vascularized areas were highly suppressed in the α-KO mice than in the Flox mice in both sexes on the 14th day after implantation (Fig. 2c). While the vascularized areas increased from day 14 to 28 in all groups of mice, the areas were significantly smaller in the male α-KO mice than in the male Flox mice at day 28. However, in the female mice of both genotypes, the areas of vascularization were of similar sizes at day 28 (Fig. 2c). Similar experiment was conducted using collagen type IV as a blood vessel-specific ECM, and the identical results were obtained as those obtained in CD31-staining (Supplementary Fig. 3).

To understand the underlying mechanism of decreased vascularized area in the male α-KO mice, parameters related to blood vessels in sponge matrices were examined in the male mice on the 14th day after implantation (Fig. 2d–g). CD31-positive blood vessels of similar sizes were detected in both genotypes (Fig. 2d). While the density of CD31-positive blood vessels was significantly lower in the α-KO mice than in the Flox mice (Fig. 2e), the difference in blood vessel density was much smaller than that in vascularized area in the comparison of the two genotypes (Fig. 2c). The number of Ki67-positive proliferating endothelial cells and the composition ratio of the CD31-positive blood vessels of various diameters were similar between the two genotypes (Fig. 2f,g). In real-time PCR analyses, among those mRNAs related to endothelial cells, the levels of mRNAs of *claudin 5* (*Cldn5*) and *cadherin 5* (*Cdh5*) were significantly suppressed at day 28 in the α-KO mice compared to that in the Flox mice (Fig. 2h). Similarly, *Cldn5*, *Cdh5* and *Cd31* mRNA expression at day 14, and *Cd31* mRNA expression at day 28 were tended to be lower in the α-KO mice compared to those in the Flox mice, respectively, while the differences between the two genotypes were not significant (Fig. 2h). Thus, the results indicate that decreased vascularized area was the most prominent phenotype among the several parameters related to blood vessels after inactivation of *Pdgfra*.

**Figure 3:**
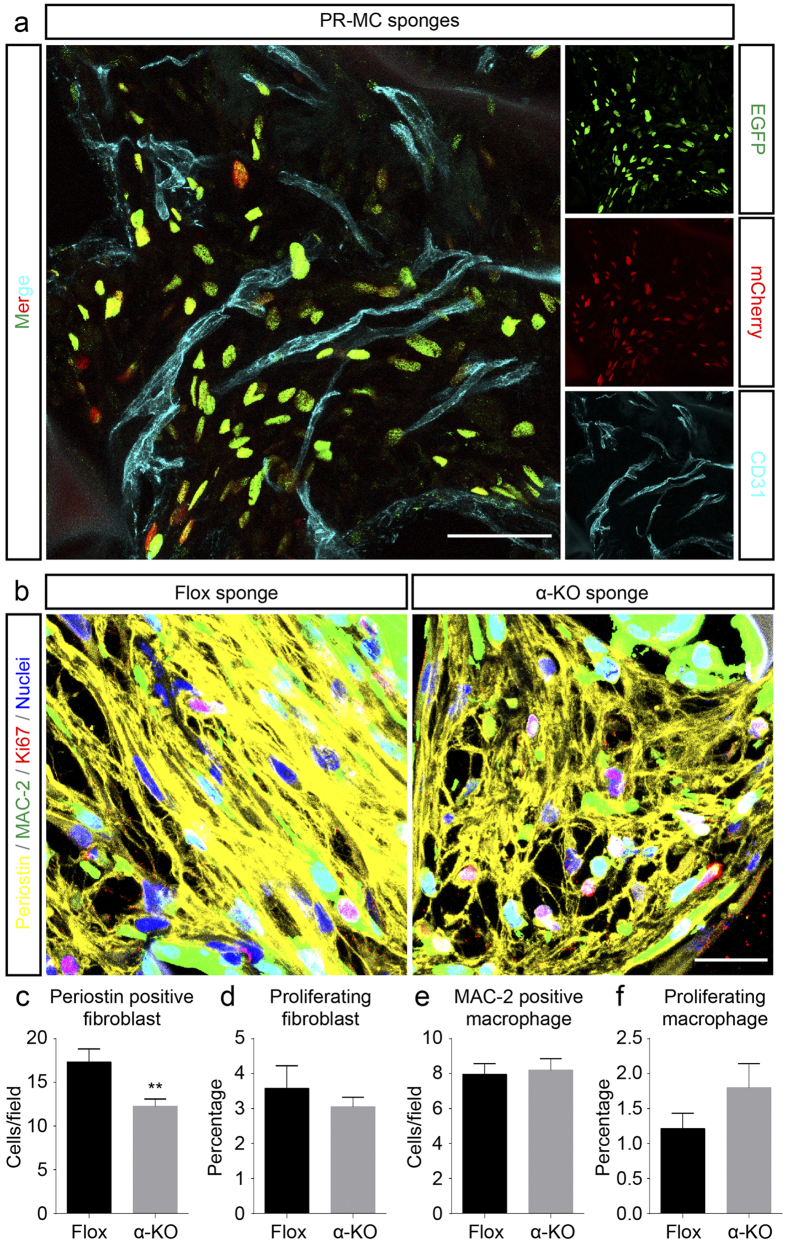
PDGFRα was not detected in blood vessels, and fibroblast recruitment is suppressed in implanted sponge in the α-KO mice. (**a**) Dual reporter assay in PR-MC mice show current activity of the PDGFRα promoter and history of tamoxifen-induced Cre activation, as EGFP (green) and mCherry (red), respectively. CD31 positive endothelial cells (cyan) are not positive for neither of these two reporter fluorescence proteins. Scale bar indicates 50 μm. (**b**) Multi-color fluorescence shows that the recruitment of periostin-positive fibroblasts (yellow) into the ingrowing connective tissues is suppressed in the α-KO mice. Macrophages (green) and Ki67-positive proliferating cells (red) are of the same level in both genotypes. Nuclei are depicted by Hoechst staining (blue). Scale bar indicates 20 μm. (**c**,**d**) Morphometrical analysis of fibroblasts. Number of periostin-positive fibroblasts is significantly less in α-KO mice than in Flox mice (**c**), but the number of periostin/Ki67-positive proliferating fibroblasts is similar between the two genotypes (**d**). (**e**,**f**) Morphometrical analysis of macrophages. Number of MAC-2 positive macrophages and MAC-2/Ki67-positive proliferating macrophages (**e**,**f**, respectively) are similar between the two genotypes.

PDGFRα was not detected within CD31-positive endothelial cells in our double-immunofluorescence (Fig. 1b). In addition to this, we further analyzed newly generated PR-MC mice to determine whether PDGFRα was expressed in vascular endothelial cells or in their precursor cells (Fig. 3a). In the PR-MC mice, EGFP-positive cells represent PDGFRα-expressing cells as EGFP is transcribed under the PDGFRα promoter. In the same mice, mCherry-positive cells represent PDGFRα-expressing cells at the time when tamoxifen was administrated, as mCherry is a reporter of Cre recombinase that is expressed under the PDGFRα promoter and is activated by tamoxifen. EGFP and mCherry were colocalized with each other, but not with CD31, in the sponge matrices implanted into PR-MC at day 14 after implantation. Furthermore, most of EGFP or mCherry-positive cells did not closely associate with vasculatures delineated by CD31 stainings. These data indicated that PDGFRα was not expressed in endothelial cells or their precursors throughout the experiment, and that the pericytes were not major population expressing PDGFRα. Together with our previous double-immunofluorescence studies (Fig. 1), the results in PR-MC mice led to the suggestion that the vascular phenotype observed in the α-KO mice is likely to be a secondary phenomenon after the *Pdgfra* inactivation that mainly occurred in fibroblasts.

### Reduced fibroblast number after PDGFRα suppression in fibroblasts

We examined the number and the mitogenic activities of infiltrating macrophages and fibroblasts into the implanted sponge matrices. To this end, the immunostaining of periostin and MAC-2, markers for fibroblasts and macrophages respectively, was conducted in combination with Ki67 as a marker of cell proliferation. In highly magnified view of multicolor immunofluorescence images, many periostin-positive fibroblasts were seen infiltrating into the sponge in the Flox mice, while they were relatively few in the α-KO mice (Fig. 3b). In contrast, MAC-2 positive macrophages were seen to infiltrate to similar extents in two genotypes (Fig. 3b). In morphometrical analysis, periostin-positive fibroblasts were slightly, but significantly, fewer in the α-KO than in the Flox mice (Fig. 3c). The number of periostin/Ki67-positive proliferating fibroblasts tended to be lower in the α-KO mice than in the Flox mice, while the differences between the two genotypes were not significant (Fig. 3d). For dissect out more detail, M phase specific marker, phospho-histone H3 (pHH3), staining was performed. Immunofluorescence and morphometrical analysis unveiled that percentage of periostin/pHH3-positive dividing fibroblasts were significantly fewer in α-KO than in Flox mice (Supplementary Fig. 4a,b). In contrast to fibroblasts, the number of MAC-2-positive macrophages and MAC-2/Ki67-positive proliferating macrophages were not significantly different between the two genotypes (Fig. 3e,f). Percentage of MAC-2/pHH3-positive dividing macrophages were also similar level in the two genotypes (Supplementary Fig. 4c). Similarly to this, M1, M2 and pan-macrophage marker genes were expressed at similar levels between the two genotypes (Supplementary Fig. 5a–f). The results show that *Pdgfra* inactivation suppressed the proliferation and infiltration of fibroblasts, but did not significantly affect those of macrophages.

### Suppression of PDGFRα decreases ECM accumulation in implanted sponges

**Figure 4:**
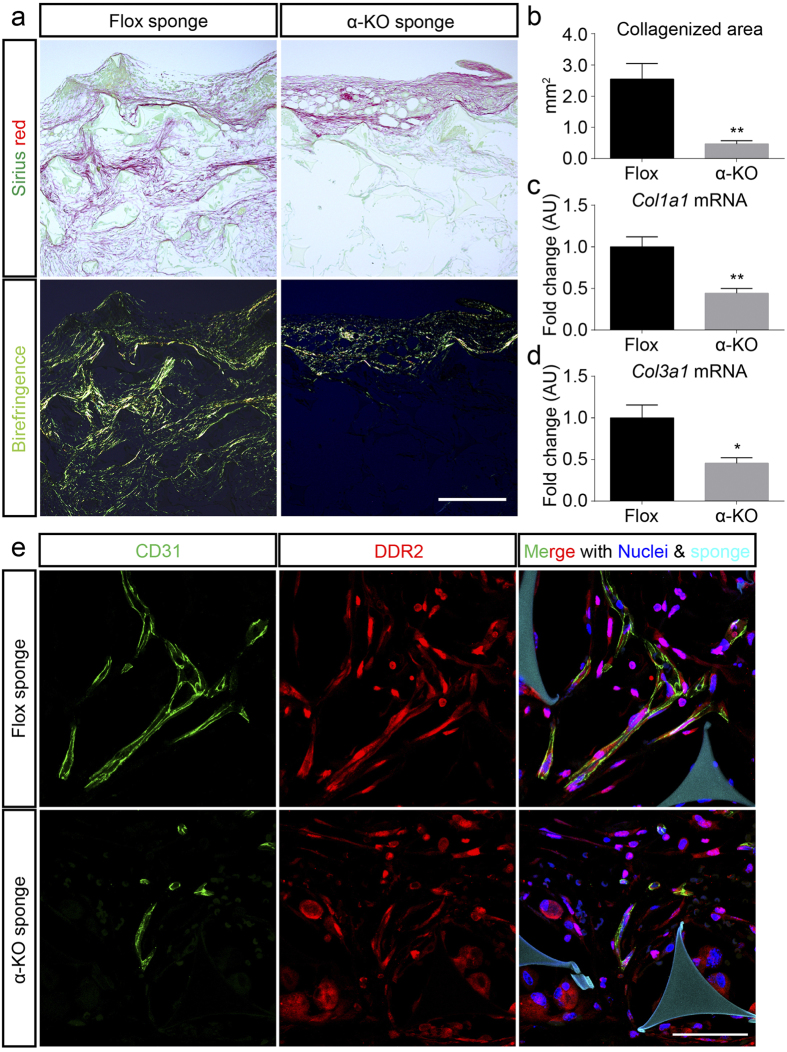
Collagen deposition is impaired in implanted sponge in α-KO. (**a**) The collagen deposition was examined in the ingrowing connective tissue by Sirius Red staining. Sirius Red staining from Flox (left) and α-KO (right) mice. Collagen is stained red under non-polarized microscope (upper row). Thick collagen fibers show strong birefringence of yellow to red color, and thin collagen shows a weak and greenish birefringence under polarized light microscope (bottom row). (**b**) Collagenized area of ingrowing connective tissue of male mice from both genotypes at day 14 was measured by quantifying polarized area of Sirius Red (mm^2^); n = 4–5 mice per group. (**c**,**d**) Real-time PCR analyses of representative ECM-related mRNAs in ingrowing connective tissue at day14 after implantation. In α-KO mice, *Col1a1*, and *Col3a1* expression levels were significantly reduced compared to that in Flox mice sponges; n = 4 per group. *p < 0.05 versus Flox mice; **p < 0.01 versus Flox mice. (**e**) In the sponges implanted in Flox mice, collagen receptor DDR2 (red) expresses in CD31 positive blood vessels (green) (upper row). Similarly, DDR2 expresses in CD31 positive blood vessels of the implanted sponges in the α-KO mice (bottom row). Nuclei are depicted by Hoechst (blue), and sponges are delineated by autofluorescence (cyan). Scale bars indicate 300 μm (**a**), and 50 μm (**e**).

Since fibroblasts, the main target of *Pdgfra* inactivation, are the principal source of ECM in connective tissue, the deposition of ECM in the ingrowing connective tissue was examined^21^. In non-polarized and polarized light microscopic views after Sirius Red staining, the amount of collagen was apparently lesser in the α-KO mice than in the Flox mice at day 14 (Fig. 4a). Within the captured polarized light microscopy images, the polarized light, indicative of collagen, was estimated to be significantly lower in the α-KO mice compared to that in the Flox mice at day 14 after implantation (Fig. 4b). In agreement with these observations, the transcript levels of *Col1a1*, and *Col3a1*, both major components of ECM in during CTR^22^,^23^, were apparently downregulated in the α-KO compared to those in the Flox mice at day 14 (Fig. 4c,d). Moreover, cultured fibroblasts isolated from α-KO mice were expressed significantly lower transcript levels of *Col1a1* and *Col3a1* than fibroblasts isolated from Flox mice (Supplementary Fig. 6a,b). Discoidin domain-containing receptor 2 (DDR2) has been shown to regulate cell-collagen interactions^24^, and endothelial cell-expressed DDR2, mediates angiogenesis^25^,^26^. In both the genotypes, DDR2 was similarly detected in CD31-positive vascular endothelial cells as well as in other CD31-negative cell types (Fig. 4e).

**Figure 5:**
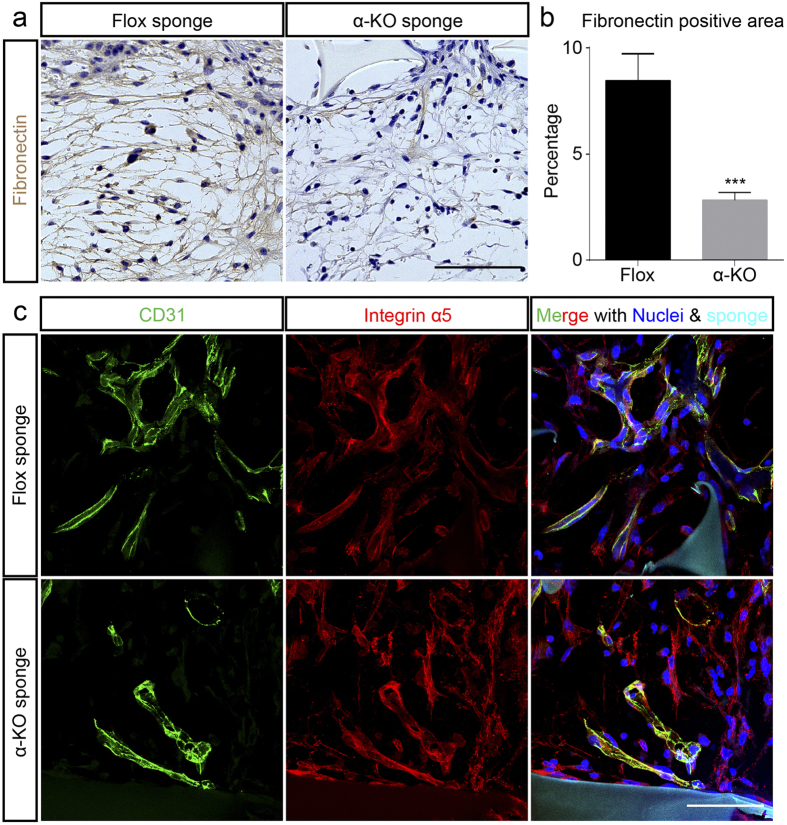
Fibronectin deposition is impaired in implanted sponge in α-KO mice. (**a**) Immunohistochemical analysis of the fibronectin deposition (brown) in connective tissue-formed sponges. Representative images of male Flox (left) and α-KO (right) mice at day 14 after implantation. α-KO mice show lesser extent of immunoreactivity to fibronectin compared to Flox mice. (**b**) In α-KO mice, the fibronectin-depositing area is significantly less than in Flox mice. ***p < 0.001 versus Flox mice. n = 4 mice per group. (**c**) In Flox mice, integrin α5 (red), a component of fibronectin receptor dimer, is expressed in CD31-positive blood vessels (green) within the sponge (upper row). Similarly, integrin α5 is expressed in CD31-positive blood vessels of ingrowing connective tissue in the α-KO mice (bottom row). Nuclei are depicted by Hoechst (blue), and sponges are visible due to the autofluorescence (cyan). Scale bars indicate 100 μm (**a**), and 50 μm (**c**).

Fibronectin is another ECM component that mediates cell adhesion and migration, and critically contributes to CTR^27^,^28^. The immunoreactivities of fibronectin in regrowing connective tissue are evidently decreased in the α-KO than in the Flox mice at day 14 (Fig. 5a). The fibronectin-positive area was significantly reduced in the α-KO than in the Flox mice in the immuno-stained sections at day 14 (Fig. 5b). *Fn1* mRNA expression was similar in both genotypes (data not shown). In agreement with *Fn1* mRNA expression in sponge, *Fn1* mRNA in fibroblasts were similar level in both genotypes (Supplementary Fig. 6c). Integrin α5, which is one of the major fibronectin receptors, was abundantly detected in CD31-positive vascular endothelial cells as well as in CD31-negative cells in both genotypes (Fig. 5c).

Both mRNA and protein expressions of collagen were decreased (Fig. 4, Supplementary Fig. 6), and the protein but not mRNA expression of fibronectin was decreased (Fig. 5, Supplementary Fig. 6). Assuming that the reduction in deposition of ECM by fibroblasts, in which, decreased collagen synthesis rather than fibronectin was hypothesized as the primary change, after gene-inactivation resulted in decreased vascularization, type I collagen was focally injected into the implanted sponge in the α-KO mice for rescue of the vascular phenotypes. However, the injection of type I collagen did not affect the CD31-positive vascularized areas in either genotype (data not shown).

### Suppression of PDGFRα decreases hepatocyte growth factor (HGF) expression level in implanted sponges

**Figure 6:**
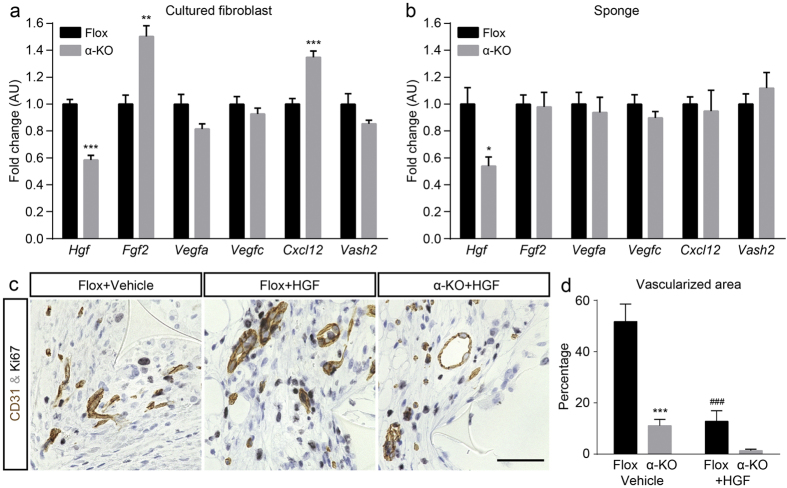
HGF expression is significantly suppressed in implanted sponges in the α-KO mice. (**a**,**b**) Real-time PCR analyses of representative angiogenic factor mRNAs, *Hgf*, *Fgf2*, *Vegfa*, *Vegfc*, *Cxcl12*, and *Vash2*. Analyses of cultured fibroblasts (**a**) and ingrowing connective tissue at day 14 (**b**) show decreased *Hgf;* n = 4−6 per group. (**c**,**d**) The rescue experiments by HGF injection were performed using Flox and α-KO mice. (**c**) Double-immunostaining of CD31 (brown) and Ki67 (black), and counterstaining with hematoxylin (pale blue) of ingrowing connective tissue at day 14 after implantation. Representative microscopic views of non-HGF-treated (vehicle-treated) Flox mice as a control (left), HGF-administrated Flox mice (middle), and HGF-administrated α-KO mice (right). Dilated blood vessels are found in the HGF-administrated groups compared to those in non-HGF-treated mice. Scale bar indicates 50 μm. (**d**) HGF administration stabilized blood vessel formation in sponges of both genotypes. ***p < 0.001 α-KO versus Flox mice; ^###^p < 0.001 non-treated Flox versus HGF treated Flox mice.

Due to the decreased vascularized areas in the α-KO mice, we examined the synthesis of representative angiogenic growth factors in cultured Flox and α-KO fibroblasts. *Hgf* mRNA expression was significantly lower in α-KO fibroblasts than in Flox cells (Fig. 6a). The abundance of *Fgf2* and *Cxcl12* transcripts was significantly higher in the α-KO fibroblasts compared to the Flox fibroblasts, while *Vegfa*, *Vegfc*, and *Vash2* levels were similar between the two genotypes (Fig. 6a). In the implanted sponge matrices, *Hgf* mRNA, but none of the other mRNAs examined, was significantly lower in the α-KO than in the Flox mice at day 14 (Fig. 6b). In support of these, VEGF-A was detected at similar extent in MAC-2-positive macrophage in both genotypes (Supplementary Fig. 5g).

Based on above data, HGF was focally injected into the implanted sponge for 14 days to rescue the decreased vascularization in the α-KO mice. Measurements of the vascularized areas in CD31-stained sections after HGF injection showed that they were largely suppressed in both genotypes (Fig. 6d). Moreover, higher magnification views of tissue from both genotypes showed that blood vessels tended to be dilated and were accompanied by increased Ki67-positive cells (Fig. 6c, Supplementary Fig. 7). Additionally, immunohistochemistry of collagen type I and fibronectin proteins showed that improvement of the ECM deposition was observed by HGF administration in α-KO mice (Supplementary Fig. 8). Thus, it seems that HGF did not enhance the revascularization rather induced the maturation of blood vessels; however, it could improve ECM deposition in connective tissues.

## Discussion

PDGFs are crucially involved in the tissue remodeling in wound healing, as well as in tumor progression. The present study, to the best of our knowledge, is the first characterization of the specific functional contributions of PDGFRα in CTR. The formation of new connective tissue was significantly delayed in sponges implanted subcutaneously when *Pdgfra* was inactivated by the Cre-loxP method. The decrease in both ECM-deposition and angiogenesis, when PDGFRα expression had been largely suppressed in the α-KO fibroblasts, were the most striking phenotypes obtained in the connective tissue growing into the sponge matrices.

PDGFRα was co-expressed along with PDGFRβ within the fibroblasts of the Flox mice, but was substantially suppressed in the α-KO mice. The expression of PDGFRα and PDGFRβ can be induced in vascular endothelial cells, and has been reported to directly contribute to the blood vessel formation^29^,^30^. However, the present study did not detect any significant expression of PDGFRα in endothelial cells and macrophages. Double-immunofluorescence studies, conducted using markers of these cells, as well as reporter assays in *Pdgfra*-preserving PR-MC mice could not detect the expression of PDGFRα in vascular endothelial cells. Accordingly, the vascular phenotypes observed in the connective tissue of the α-KO mice are likely to be due to the disturbed function of fibroblasts induced by inactivation of *Pdgfra*.

Fibroblasts are the principal source of ECM in connective tissue^21^,^31^,^32^, and hence were the primary target of *Pdgfra* inactivation in the present study. Collagens and fibronectin are essential components of the ECM during CTR. As the results show, accumulation of collagen and fibronectin was largely suppressed in the ingrowing connective tissue in the α-KO mice, suggesting that the PDGFRα importantly mediates ECM deposition during CTR. In the α-KO mice, fibroblast cell number in sponges was decreased, and collagen synthesis in fibroblasts was suppressed; both of these findings were supposed to additively contribute to the decreased collagen deposition as a direct sequence of *Pdgfra* inactivation. On the other hand, whereas the deposition of fibronectin was largely suppressed in α-KO mice, *Fn1* expression was similar between two genotypes both *in vivo* and *in vitro* analyses. These data suggest that majority of the fibronectin in sponges is derived from plasma as previously reported^33^. Furthermore, the decreased collagen deposition may be the underlying reason of less fibronectin deposition which binds to collagen *via* the collagen-binding domain^34^ in α-KO mice.

Collagens and fibronectin promote angiogenesis by preparing the matrix framework for new vessel formation^35^,^36^ and by supporting endothelial cell proliferation, survival, and migration^37^. In the α-KO mice, accumulation of collagen and fibronectin was largely suppressed, in accordance with the substantial decrease of angiogenesis. On the other hand, the endothelial cells preserved DDR2 and integrin α5, the receptors for these ECM components in the α-KO mice^26^,^38^,^39^. Thus, altered intrinsic nature of endothelial cells were not indicated to be the primary cause of decreased neovascularization. Stimulation of angiogenesis has been shown to enhance the rate of healing, particularly in impaired healing models such as diabetic subjects^40^,^41^, as well as to promote tumor progression^18^. Accordingly, the present observations on ECM deposition and neovascularization indicate that the PDGFRα could be an important target to regulate tissue remodeling in diverse diseases.

HGF is a mitogen of endothelial cells and connective tissue cells^42^. Mesenchymal cells promote angiogenesis by secreting angiogenic growth factors and cytokines, among which PDGFRα mediates the expression of fibroblast growth factor 2 (FGF2)-induced HGF and vascular endothelial growth factor A (VEGF-A) in these cells^43^. Along this line, real-time PCR analyses indicated that *Hgf*, but not the other angiogenic factors examined, was significantly decreased in both *Pdgfra*-inactivated fibroblasts and in ingrowing connective tissue of α-KO mice, compared to those in *Pdgfra*-preserving controls. Based on these observations, HGF was focally applied to the implanted sponge to improve ingrowth of connective tissue. Applied HGF enhanced the fibroblastic cell proliferation and the deposition of collagen type I and fibronectin in the ingrowing connective tissue. Furthermore, HGF induced the vascular endothelial cell proliferation and vascular dilatation that is one of the important modes of actions of HGF to induce angiogenesis^44^. Accordingly, it was shown that HGF induced by PDGFRα was involved in the ECM and vascular remodeling. However, HGF-treatment did not induce ingrowth of blood vessels in Flox or α-KO mice. Since HGF and other growth factors associate with ECM through growth factor-binding sites^45^, it is possible that a gradient of growth factor concentration is necessary for inducing neovascularization and such a gradient could not be established in absence of the ECM.

In conclusion, we have shown, by using a sponge-implantation mouse model, that PDGFRα mediates CTR. Angiogenesis and fibroplasia are interdependent and crucial components of CTR. The reduction in both ECM formation and HGF production by *Pdgfra*-inactivated fibroblasts is likely to be the main reason for the substantial delay in angiogenesis and fibroplasia in the α-KO mice; however, further studies will be required to understand the detailed mechanism of PDGFRα action, and its interaction with other molecular determinants of CTR. Furthermore, the results suggest that PDGFRα is an important target to regulate tissue remodeling in diverse diseases.

## Methods

### Generation of conditional gene-inactivation and transgenic mice

Mutant mice, harboring the *Pdgfra* gene in which the exons 4 and 5 were flanked by two loxP sequences (*Pdgfra*^*flox/flox*^), were generated as follows: Briefly, BAC genomic clone (RP24-148N4), originating from the DNA of C57BL/6 mice and containing the *Pdgfra* gene, was obtained from the BACPAC Resource Center CHORI (Oakland, CA, USA). The targeting vector included a DNA fragment containing a *loxP* sequence and a *pgk-neo* cassette flanked by two Flp recognition target (*frt*) sites^46^ inserted into pMC1DTABGHA that derived from pMC1DTApAM^47^ after replacing the pgk-pA signal sequence with the bovine growth hormone-derived pA signal. The embryonic stem cell line RENKA, derived from the C57BL/6N strain^48^, was used and the targeting vector was electroporated as described previously^49^. The resultant male chimeric mice were crossed with female CAG-FLPe deletion mutant mice^50^ to remove the *pgk-neo* selection cassette and obtain the heterozygous *Pdgfra*^*flox*/+^ strain. Genotyping was performed by PCR using the following primers: 5′-ATGCCAAACTCTGCCTGATTGA-3′ and 5′-CTCACGGAACCCCCACAAC-3′. The Flox mice were crossbred with *chicken β-actin-promoter*/*CMV-enhancer*-driven *Cre*-transgenic mice harboring tamoxifen-inducible *Cre recombinase* (*Cre-ER*^+/−^; *CAGGCre-ER*^+/−^; Jackson Laboratories, Bar Harbor, ME, USA)^51^. The resultant offspring harboring the *Pdgfra*^*flox/flox*^*;Cre-ER*^+/−^, and those harboring *Pdgfra*^*flox/flox*^ were respectively used as systemic *Pdgfra*-KO (α-KO) mice and as *Pdgfra*-preserving controls (Flox mice) after tamoxifen administration, as described below. Transgenic mice harboring the transgene, which includes the *Pdgfra* promoter region in combination with *cre recombinase* gene in tandem with *enhanced green fluorescent protein* (*Egfp*) gene inserted by *internal ribosome entry site (IRES)* sequence, were generated as follows. Briefly, BAC genomic clone (RP24-148N4), originating from the DNA of C57BL/6 mice and containing the *Pdgfra* gene, was obtained from the BACPAC Resources Center CHORI (Oakland, CA). The targeting vector included a DNA fragment of an improved tamoxifen-inducible *Cre recombinase* (*CreERT2*) and *Egfp,* both of which were connected by the *IRES* sequence, and pBigTDEST^52^, which carries a triple repeat of SV40 polyadenylation signal sequence. The embryonic stem cell line TT2^52^, derived from the F1 embryo produced by a cross between a C57BL/6 female and a CBA male, was used and the targeting vector was electroporated as described previously^49^. The obtained male chimeric mice were crossed with C57BL/6 mice to obtain the heterozygous *Pdgfra-CreERT2-Egfp* (PR) strain. Genotyping was performed by PCR using the following primers: 5′-GGCAAGCTGACCCTGAAGTTCATCTGCACC-3′ and 5′-ATCGCGCTTCTCGTTGGGGTCTTTGCTCAG-3′. The PR mice were crossbred with *R26R*^*H2B-mCherry/*+^ mice^53^ and the resulting offsprings, which harbored *Pdgfra-CreERT2-Egfp*^+/−^*;R26R*^*H2B-mCherry/*+^ (PR-MC mice), were treated with tamoxifen and used as a reporter of *Pdgfra* gene inactivation within PDGFRα-expressing cells. All the mice were housed at 25 °C with a 12 h light/12 h dark cycle and free access to pellet chow and water. All experimental animal procedures were approved by the Committee for Institutional Animal Care and Use at the University of Toyama (Toyama, Japan), and experiments were carried out in accordance with the approved guidelines.

### Sponge implantation and suppression of PDGFRα expression

Circular sponge discs were implanted into 4–5-month-old mutant mice under deep anesthesia as described previously^54^. Briefly, circular sponge discs (5 mm thick, 1.3 cm in diameter) were formed from a sheet of polyurethane form (Komeri, Niigata, Japan) using appropriate-sized punch. The discs were soaked in 70% ethanol for 3 hours and the rinsed in sterile distilled water. After drying at reduced pressure, all discs were autoclaved before implantation. Tamoxifen (Sigma-Aldrich, St. Louis, MO) was orally administered once a day (225 mg/kg body weight) for 5 consecutive days^55^, starting on days 0 and 14 after sponge implantation. At days 14 and 28 after implantation, connective tissue-formed sponges were excised together with the enclosed sponge implants under deep anesthesia. The obtained tissues were used for real-time PCR, western blotting, and histological studies.

### Real-time PCR

Total RNA isolation and quantitative real-time PCR analysis were performed as described previously^56^,^57^. Briefly, for real-time PCR, performed with a Thermal Cycler Dice Real Time System TP800 (Takara, Kyoto, Japan), cDNAs were diluted 1:25 in the reaction mixture consisting of SYBR Premix EX Taq II (Takara). The real-time PCR program consisted of hot start enzyme activation at 95 °C for 10 s, 40 cycles of amplification at 95 °C for 10 s and 60 °C for 40 s. Finally, to obtain the dissociation curve, a final cycle was performed at 95 °C for 1 min, 60 °C for 30 s, and then 95 °C for 10 s. For data analysis, mouse β*-actin* (*Actb*) or *glyceraldehyde-3-phosphate dehydrogenase* (*Gapdh*) housekeeping genes was used as internal control. Induction values were calculated using analysis software. Primer sequences are available upon request from the Takara Bio Inc. website (http://www.takara-bio.co.jp/).

### Immunohistochemistry and immunofluorescence staining of paraffin-embedded or frozen sections

For paraffin section preparation, the extracted sponges and surrounding connective tissues were immediately fixed with 4% paraformaldehyde in 0.1 M phosphate buffer (pH 7.4), dehydrated with a graded series of ethanol solutions, and embedded in paraffin. The sections (5 μm in thickness) were subjected to either hematoxylin and eosin (H&E) staining or immunostaining. For frozen section preparation, the extracted sponge and surrounding connective tissue were immediately fixed with 1% paraformaldehyde in 0.1 M phosphate buffer (pH 7.4) at 4 °C overnight, and then transferred to phosphate-buffered saline (PBS). Tissues were submerged in 30% PBS-buffered sucrose, and then the entire tissue was embedded in optimal cutting temperature (OCT) compound (Sakura Finetek Japan, Tokyo, Japan) and frozen at −20 °C until solidified. The frozen sections, each 30 μm in thickness, were cut on a CM3000 cryostat (Leica Microsystems, Wetzlar, Germany). Immunohistochemistry and immunofluorescence staining was performed as described previously^56^,^58^. Briefly, the sections were incubated at 4 °C overnight with the following primary antibodies: goat polyclonal anti-PDGFRα (1:100; Neuromics, Edina, MN), rabbit monoclonal anti-PDGFRβ (1:100; Epitomics, Burlingame, CA), rat monoclonal anti-F4/80 (1:500; Bio-Rad Laboratories, Hercules, CA), rat monoclonal anti-CD31 (1:500; Dianova, Hamburg, Germany), rabbit polyclonal anti-Periostin (1:100; Abcam, Cambridge, MA), biotinylated rat monoclonal anti-MAC-2 (1:10; BioLegend, San Diego, CA), rat monoclonal anti-Ki67 (1:100; eBioscience, San Diego, CA), rabbit polyclonal anti-GFP (1:100; Frontier Institute, Hokkaido, Japan), rabbit polyclonal anti-VEGF-A (1:50; Merck Millipore, Billerica, MA), rabbit polyclonal anti-collagen type I (1:250; Abcam), mouse monoclonal anti-fibronectin (1:100; Abcam), rabbit polyclonal anti-collagen type IV (1:500; Merck Millipore), and rabbit monoclonal anti-phospho-histone H3 conjugated with Alexa-Fluor 488 (1:25; Cell Signaling, Danvers, MA). Colorimetric immunostaining was done using the appropriate Histofine Simple Stain Mice System (NICHIREI BIOSCIENCES, Tokyo, Japan) and 3,3**′**-diaminobenzidine tetrahydrochloride (DAB, Dako, Glostrup, Denmark) reaction. The nuclei were counterstained with hematoxylin. For immunofluorescence, the Alexa-Fluor488-, Alexa-Fluor568-, or Alexa-Fluor633-conjugated secondary antibodies (Life Technologies Corporation, Carlsbad, CA) were used at 1:500 dilutions. Streptavidin conjugated with Alexa-Fluor488 or Alexa-Fluor568 was used for the biotinylated primary antibody. Nuclei were stained with Hoechst 33258 (Nacalai Tesque, Koto, Japan). Images were captured by a microscopy system (BX 51; Olympus, Tokyo, Japan) connected to a digital camera (DP70; Olympus) or BIOREVO BZ-9000 microscope (Keyence, Osaka, Japan), and were processed using Photoshop software (version 7.0; Adobe, San Jose, CA). Immunofluorescence staining was imaged by a confocal microscope (TCS-SP5; Leica Microsystems). Montages were created using Photoshop software.

### Image analysis and quantification

The area occupied by blood vessels in sagittal cross-sections of excised sponges was analyzed in CD31- or collagen type IV-stained sections. We defined the area encircled by the tip of CD31- or collagen type IV-positive cells and the border of sponge matrices as the vascularized area. The percentages of vascularized area were calculated by following equation; {(area of sponge matrix border)−(area of vascular tip line)}/(area of sponge matrix border)×100 = (vascularized area). The numbers of CD31-positive vascular endothelial cells were determined within five randomly selected representative views in vascularized area at high power field (×600 magnification) in a section. To determine the collagen fiber content within the halves of the sponge, the tissues were stained with Sirius Red. In Sirius Red-stained sections observed under polarization, thick collagen fibers were strongly birefringent and yellow to red, whereas thin collagen fibers were weakly birefringent and greenish^59^. Polarized images were obtained by a microscopy system (BX 51; Olympus) connected to a digital camera (DP70; Olympus). The birefringent areas within cross-sections of sponges were quantified by VH Analyzer software (VH-H1A5; Keyence). Morphological and morphometric analyses were performed using a BIOREVO BZ-9000 microscope (Keyence) and BZ-II Analyzer software (Keyence). For the fibronectin analysis, fibronectin positive areas were normalized by quantified cell areas and analyzed with the BZ-II Analyzer software (Keyence).

### Western blot analyses

Tissue preparation and blotting were performed as described previously^56^,^57^,^58^. Membranes were incubated with the following primary antibodies: rabbit polyclonal anti-PDGFRα (1:1000; Santa Cruz Biotechnology, Santa Cruz, CA); rabbit polyclonal anti-PDGFRβ (1:500; Merck Millipore), and mouse monoclonal anti-GAPDH (1:5000; Merck Millipore) at 4 °C overnight. The immunoreactive bands of target proteins were quantified by VH Analyzer software (VH-H1A5, Keyence, Osaka, Japan), and normalized to the GAPDH protein band.

### Cell culture

Skins were harvested from postnatal day 1 (P1)-P3 pups of *Pdgfra*^*flox/flox*^*;Cre-ER*^+/−^ and *Pdgfra*^*flox/flox*^ mice under the deep anesthesia, incubated with Dulbecco’s modified eagle media (D-MEM, Nacalai Tesque) containing 0.3% collagenase (Sigma-Aldrich) in 4 °C for 48 h. The dermis was separated from the epidermis in D-MEM containing 10% fetal bovine serum (FBS), and minced by scissors and triturated by pipetting. The cell suspension filtered through 100-μm nylon mesh (BD Bioscience, San Jose, CA) was centrifuged; the pellet was then resuspended in DMEM containing 10% FBS and seeded in 10 cm plastic dishes. After several passages, cells were stored in −80 °C until usage for experiments. Both of fibroblasts were seeded on 6-well plate, and were cultured with D-MEM containing 10% FBS for 24 h. After the cells were subjected with 1 μM of 4-hydroxytamoxifen (4-OHT, Sigma-Aldrich) for deleting of Floxed allele for 48 h, resulting α-KO and Flox were generated.

### Collagen type I injection

To clarify the role of PDGFRα-induced expression of collagen type I, high density collagen type I, extracted from rat tail (30 μg dissolved in 100 μl PBS with 0.023 M acetic acid and 0.023 M sodium hydroxide; BD Bioscience), was focally injected into sponge implants once a day on days 3 and 7 after implantation. The implanted sponges were removed on day 14, and evaluated histologically.

### Hepatocyte Growth Factor (HGF) injection

To clarify the role of PDGFRα-induced HGF expression, the activated form of human HGF (kindly provided by Professor Kitamura, Tokyo Institute of Technology, School and Graduate School of Bioscience and Biotechnology) was topically injected at concentration of 1 μg in 50 μL PBS, with 0.1% BSA, into sponge implants once a day from days 2 to 13 after implantation. The implanted sponges were removed on day 14, and histologically evaluated.

### Statistics

Statistical significance was determined using Student’s t-test, or one- or two-way analysis of variance (ANOVA) with Tukey’s multiple comparisons as a post-hoc analysis for ANOVA. P values less than 0.05 were considered statistically significant. Graphs were drawn using GraphPad Prism 6 (GraphPad Software, Inc., La Jolla, CA). Quantified data are presented as mean ± SEM.

## Additional Information

**How to cite this article**: Horikawa, S. *et al.* PDGFRα plays a crucial role in connective tissue remodeling. *Sci. Rep.* **5**, 17948; doi: 10.1038/srep17948 (2015).

## Supplementary Material

Supplementary Figures

## References

[b1] Donovan J., Abraham D. & Norman J. Platelet-derived growth factor signaling in mesenchymal cells. Front Biosci (Landmark Ed) 18, 106–119 (2013). 23276912 10.2741/4090

[b2] Crawford Y. *et al.* PDGF-C mediates the angiogenic and tumorigenic properties of fibroblasts associated with tumors refractory to anti-VEGF treatment. Cancer Cell 15, 21–34 (2009). 19111878 10.1016/j.ccr.2008.12.004

[b3] Papanas N. & Maltezos E. Growth factors in the treatment of diabetic foot ulcers: new technologies, any promises? Int J Low Extrem Wounds 6, 37–53 (2007). 17344201 10.1177/1534734606298416

[b4] Singer A. J. & Clark R. A. Cutaneous wound healing. N Engl J Med 341, 738–746 (1999). 10471461 10.1056/NEJM199909023411006

[b5] Barrientos S., Stojadinovic O., Golinko M. S., Brem H. & Tomic-Canic M. Growth factors and cytokines in wound healing. Wound Repair Regen 16, 585–601 (2008). 19128254 10.1111/j.1524-475X.2008.00410.x

[b6] Andrae J., Gallini R. & Betsholtz C. Role of platelet-derived growth factors in physiology and medicine. Genes Dev 22, 1276–1312 (2008). 18483217 10.1101/gad.1653708 PMC2732412

[b7] Heldin C. H. & Westermark B. Mechanism of action and *in vivo* role of platelet-derived growth factor. Physiol Rev 79, 1283–1316 (1999). 10508235 10.1152/physrev.1999.79.4.1283

[b8] Crosby J. R., Tappan K. A., Seifert R. A. & Bowen-Pope D. F. Chimera analysis reveals that fibroblasts and endothelial cells require platelet-derived growth factor receptorbeta expression for participation in reactive connective tissue formation in adults but not during development. Am J Pathol 154, 1315–1321 (1999). 10329583 10.1016/s0002-9440(10)65384-9 PMC1866587

[b9] Gao Z. *et al.* Deletion of the PDGFR-beta gene affects key fibroblast functions important for wound healing. J Biol Chem 280, 9375–9389 (2005). 15590688 10.1074/jbc.M413081200

[b10] Rajkumar V. S. *et al.* Platelet-derived growth factor-beta receptor activation is essential for fibroblast and pericyte recruitment during cutaneous wound healing. Am J Pathol 169, 2254–2265 (2006). 17148686 10.2353/ajpath.2006.060196 PMC1762470

[b11] Nagai M. K. & Embil J. M. Becaplermin: recombinant platelet derived growth factor, a new treatment for healing diabetic foot ulcers. Expert Opin Biol Ther 2, 211–218 (2002). 11849120 10.1517/14712598.2.2.211

[b12] Iwayama T. & Olson L. E. Involvement of PDGF in fibrosis and scleroderma: recent insights from animal models and potential therapeutic opportunities. Curr Rheumatol Rep 15, 304 (2013). 23307576 10.1007/s11926-012-0304-0 PMC5570472

[b13] Olson L. E. & Soriano P. Increased PDGFRalpha activation disrupts connective tissue development and drives systemic fibrosis. Dev Cell 16, 303–313 (2009). 19217431 10.1016/j.devcel.2008.12.003 PMC2664622

[b14] Plasari G. *et al.* Nuclear factor I-C links platelet-derived growth factor and transforming growth factor beta1 signaling to skin wound healing progression. Mol Cell Biol 29, 6006–6017 (2009). 19752192 10.1128/MCB.01921-08 PMC2772573

[b15] Hou X. *et al.* PDGF-CC blockade inhibits pathological angiogenesis by acting on multiple cellular and molecular targets. Proc Natl Acad Sci USA 107, 12216–12221 (2010). 20566880 10.1073/pnas.1004143107 PMC2901428

[b16] Li X. *et al.* PDGF-C is a new protease-activated ligand for the PDGF alpha-receptor. Nat Cell Biol 2, 302–309 (2000). 10806482 10.1038/35010579

[b17] Li X. *et al.* Revascularization of ischemic tissues by PDGF-CC via effects on endothelial cells and their progenitors. J Clin Invest 115, 118–127 (2005). 15630451 10.1172/JCI19189 PMC535797

[b18] Li X. *et al.* VEGF-independent angiogenic pathways induced by PDGF-C. Oncotarget 1, 309–314 (2010). 20871734 10.18632/oncotarget.141 PMC2944232

[b19] Martin S. & Murray J. C. Angiogenesis protocols. 2nd edn, Vol. 467 (Humana Press Springer, 2009).

[b20] Siemerink M. J., Klaassen I., Van Noorden C. J. & Schlingemann R. O. Endothelial tip cells in ocular angiogenesis: potential target for anti-angiogenesis therapy. J Histochem Cytochem 61, 101–115 (2013). 23092791 10.1369/0022155412467635 PMC3636692

[b21] Kendall R. T. & Feghali-Bostwick C. A. Fibroblasts in fibrosis: novel roles and mediators. Front Pharmacol 5, 123 (2014). 24904424 10.3389/fphar.2014.00123 PMC4034148

[b22] Reinke J. M. & Sorg H. Wound repair and regeneration. Eur Surg Res 49, 35–43 (2012). 22797712 10.1159/000339613

[b23] Widgerow A. D. Cellular/extracellular matrix cross-talk in scar evolution and control. Wound Repair Regen 19, 117–133 (2011). 21362079 10.1111/j.1524-475X.2010.00662.x

[b24] Vogel W., Gish G. D., Alves F. & Pawson T. The discoidin domain receptor tyrosine kinases are activated by collagen. Mol Cell 1, 13–23 (1997). 9659899 10.1016/s1097-2765(00)80003-9

[b25] Marquez J. & Olaso E. Role of discoidin domain receptor 2 in wound healing. Histol Histopathol 29, 1355–1364 (2014). 24781958 10.14670/HH-29.1355

[b26] Zhang S. *et al.* A host deficiency of discoidin domain receptor 2 (DDR2) inhibits both tumour angiogenesis and metastasis. J Pathol 232, 436–448 (2014). 24293323 10.1002/path.4311

[b27] Stoffels J. M., Zhao C. & Baron W. Fibronectin in tissue regeneration: timely disassembly of the scaffold is necessary to complete the build. Cell Mol Life Sci 70, 4243–4253 (2013). 23756580 10.1007/s00018-013-1350-0 PMC11113129

[b28] Zhu J. & Clark R. A. Fibronectin at select sites binds multiple growth factors and enhances their activity: expansion of the collaborative ECM-GF paradigm. J Invest Dermatol 134, 895–901 (2014). 24335899 10.1038/jid.2013.484 PMC3961531

[b29] Marx M., Perlmutter R. A. & Madri J. A. Modulation of platelet-derived growth factor receptor expression in microvascular endothelial cells during *in vitro* angiogenesis. J Clin Invest 93, 131–139 (1994). 7506710 10.1172/JCI116936 PMC293745

[b30] Nissen L. J. *et al.* Angiogenic factors FGF2 and PDGF-BB synergistically promote murine tumor neovascularization and metastasis. J Clin Invest 117, 2766–2777 (2007). 17909625 10.1172/JCI32479 PMC1994630

[b31] Lepisto J. *et al.* Effects of homodimeric isoforms of platelet-derived growth factor (PDGF-AA and PDGF-BB) on wound healing in rat. J Surg Res 53, 596–601 (1992). 1494293 10.1016/0022-4804(92)90260-7

[b32] Xu J. & Clark R. A. Extracellular matrix alters PDGF regulation of fibroblast integrins. J Cell Biol 132, 239–249 (1996). 8567727 10.1083/jcb.132.1.239 PMC2120701

[b33] Moretti F. A. *et al.* A major fraction of fibronectin present in the extracellular matrix of tissues is plasma-derived. J Biol Chem 282, 28057–28062 (2007). 17644525 10.1074/jbc.M611315200

[b34] Kubow K. E. *et al.* Mechanical forces regulate the interactions of fibronectin and collagen I in extracellular matrix. Nat Commun 6, 8026 (2015). 26272817 10.1038/ncomms9026 PMC4539566

[b35] Ignotz R. A. & Massague J. Transforming growth factor-beta stimulates the expression of fibronectin and collagen and their incorporation into the extracellular matrix. J Biol Chem 261, 4337–4345 (1986). 3456347

[b36] Oberringer M., Meins C., Bubel M. & Pohlemann T. *In vitro* wounding: effects of hypoxia and transforming growth factor beta1 on proliferation, migration and myofibroblastic differentiation in an endothelial cell-fibroblast co-culture model. J Mol Histol 39, 37–47 (2008). 17786573 10.1007/s10735-007-9124-3

[b37] Sottile J. Regulation of angiogenesis by extracellular matrix. Biochim Biophys Acta 1654, 13–22 (2004). 14984764 10.1016/j.bbcan.2003.07.002

[b38] Senger D. R. *et al.* The alpha(1)beta(1) and alpha(2)beta(1) integrins provide critical support for vascular endothelial growth factor signaling, endothelial cell migration, and tumor angiogenesis. Am J Pathol 160, 195–204 (2002). 11786413 10.1016/s0002-9440(10)64363-5 PMC1867136

[b39] Wilson S. H. *et al.* Fibronectin fragments promote human retinal endothelial cell adhesion and proliferation and ERK activation through alpha5beta1 integrin and PI 3-kinase. Invest Ophthalmol Vis Sci 44, 1704–1715 (2003). 12657612 10.1167/iovs.02-0773

[b40] Galiano R. D. *et al.* Topical vascular endothelial growth factor accelerates diabetic wound healing through increased angiogenesis and by mobilizing and recruiting bone marrow-derived cells. Am J Pathol 164, 1935–1947 (2004). 15161630 10.1016/S0002-9440(10)63754-6 PMC1615774

[b41] Romano Di Peppe S. *et al.* Adenovirus-mediated VEGF(165) gene transfer enhances wound healing by promoting angiogenesis in CD1 diabetic mice. Gene Ther 9, 1271–1277 (2002). 12224009 10.1038/sj.gt.3301798

[b42] Kaga T. *et al.* Hepatocyte growth factor stimulated angiogenesis without inflammation: differential actions between hepatocyte growth factor, vascular endothelial growth factor and basic fibroblast growth factor. Vascul Pharmacol 57, 3–9 (2012). 22361334 10.1016/j.vph.2012.02.002

[b43] Tsutsumi N. *et al.* Essential role of PDGFRalpha-p70S6K signaling in mesenchymal cells during therapeutic and tumor angiogenesis *in vivo*: role of PDGFRalpha during angiogenesis. Circ Res 94, 1186–1194 (2004). 15059936 10.1161/01.RES.0000126925.66005.39

[b44] Beilmann M., Birk G. & Lenter M. C. Human primary co-culture angiogenesis assay reveals additive stimulation and different angiogenic properties of VEGF and HGF. Cytokine 26, 178–185 (2004). 15149635 10.1016/j.cyto.2004.03.003

[b45] Hynes R. O. The extracellular matrix: not just pretty fibrils. Science 326, 1216–1219 (2009). 19965464 10.1126/science.1176009 PMC3536535

[b46] Takeuchi T. *et al.* Flp recombinase transgenic mice of C57BL/6 strain for conditional gene targeting. Biochem Biophys Res Commun 293, 953–957 (2002). 12051751 10.1016/S0006-291X(02)00321-2

[b47] Kitayama K. *et al.* Purkinje cell-specific and inducible gene recombination system generated from C57BL/6 mouse ES cells. Biochem Biophys Res Commun 281, 1134–1140 (2001). 11243853 10.1006/bbrc.2001.4492

[b48] Fukaya M. *et al.* Abundant distribution of TARP gamma-8 in synaptic and extrasynaptic surface of hippocampal neurons and its major role in AMPA receptor expression on spines and dendrites. Eur J Neurosci 24, 2177–2190 (2006). 17074043 10.1111/j.1460-9568.2006.05081.x

[b49] Miya K. *et al.* Serine racemase is predominantly localized in neurons in mouse brain. J Comp Neurol 510, 641–654 (2008). 18698599 10.1002/cne.21822

[b50] Kanki H., Suzuki H. & Itohara S. High-efficiency CAG-FLPe deleter mice in C57BL/6J background. Exp Anim 55, 137–141 (2006). 16651697 10.1538/expanim.55.137

[b51] Hayashi S. & McMahon A. P. Efficient recombination in diverse tissues by a tamoxifen-inducible form of Cre: a tool for temporally regulated gene activation/inactivation in the mouse. Dev Biol 244, 305–318 (2002). 11944939 10.1006/dbio.2002.0597

[b52] Yagi T. *et al.* A novel ES cell line, TT2, with high germline-differentiating potency. Anal Biochem 214, 70–76 (1993). 8250257 10.1006/abio.1993.1458

[b53] Abe T. *et al.* Establishment of conditional reporter mouse lines at ROSA26 locus for live cell imaging. Genesis 49, 579–590 (2011). 21445964 10.1002/dvg.20753

[b54] Majima M. *et al.* Significant roles of inducible cyclooxygenase (COX)-2 in angiogenesis in rat sponge implants. Jpn J Pharmacol 75, 105–114 (1997). 9414024 10.1254/jjp.75.105

[b55] Tokunaga A. *et al.* PDGF receptor beta is a potent regulator of mesenchymal stromal cell function. J Bone Miner Res 23, 1519–1528 (2008). 18410236 10.1359/jbmr.080409

[b56] Shen J. *et al.* PDGFR-beta as a positive regulator of tissue repair in a mouse model of focal cerebral ischemia. J Cereb Blood Flow Metab 32, 353–367 (2012). 21952111 10.1038/jcbfm.2011.136 PMC3272602

[b57] Yamamoto S. *et al.* Inflammation-induced endothelial cell-derived extracellular vesicles modulate the cellular status of pericytes. Sci Rep 5, 8505 (2015). 25687367 10.1038/srep08505 PMC4330530

[b58] Yamamoto S. *et al.* Essential role of Shp2-binding sites on FRS2alpha for corticogenesis and for FGF2-dependent proliferation of neural progenitor cells. Proc Natl Acad Sci USA 102, 15983–15988 (2005). 16239343 10.1073/pnas.0507961102 PMC1276098

[b59] Souza B. R., Santos J. S. & Costa A. M. Blockade of beta1- and beta2-adrenoceptors delays wound contraction and re-epithelialization in rats. Clin Exp Pharmacol Physiol 33, 421–430 (2006). 16700874 10.1111/j.1440-1681.2006.04383.x

