# Supplementary material for: PDGFRα plays a crucial role in connective tissue remodeling
Source: Sci Rep. 2015 Dec 7;5:17948. doi: 10.1038/srep17948 (PMC4671150; doi:10.1038/srep17948)
Supplement: Supplementary Figures [file srep17948-s1.pdf]

Supplementary Information

PDGFRα plays a crucial role in connective tissue remodeling

Shinjiro Horikawa[1,2], Yoko Ishii[1], Takeru Hamashima[1], Seiji Yamamoto[1,*], Hisashi Mori[3], Toshihiko Fujimori[4], Jie Shen[1], Ran Inoue[3], Hirofumi Nishizono[5], Hiroshi Itoh[6], Masataka Majima[7], David Abraham[8], Toshio Miyawaki[2,9], and Masakiyo Sasahara[1]

[1]Department of Pathology, Graduate School of Medicine and Pharmaceutical Sciences, University of Toyama, Toyama 930-0194, Japan
[2]Department of Pediatrics, Graduate School of Medicine and Pharmaceutical Sciences, University of Toyama, Toyama 930-0194, Japan
[3]Department of Molecular Neuroscience, Graduate School of Medicine and Pharmaceutical Sciences, University of Toyama, Toyama 930-0194, Japan
[4]Division of Embryology, National Institute for Basic Biology, Okazaki 444-8787, Japan
[5]Division of Animal Experimental Laboratory, Life Science Research Center, University of Toyama, Toyama 930-0194, Japan
[6]Department of Molecular Pathology, Graduate School of Medicine, Yamaguchi University, Ube 755-8505, Japan
[7]Department of Pharmacology, School of Medicine, Kitasato University, Kanagawa 252-0374, Japan
[8]Division of Medicine, University College London, Royal Free Campus, London, NW3 2PF, UK
[9]Deceased, we dedicate this article to the memory of a brilliant and beloved colleague

*Correspondence to: Seiji Yamamoto, Ph.D., Department of Pathology, Graduate School of Medicine and Pharmaceutical Sciences, University of Toyama, Toyama 930-0152, Japan
Tel: +81-076-434-2281, Fax: +81-076-434-5016, E-mail: seiyama@med.u-toyama.ac.jp

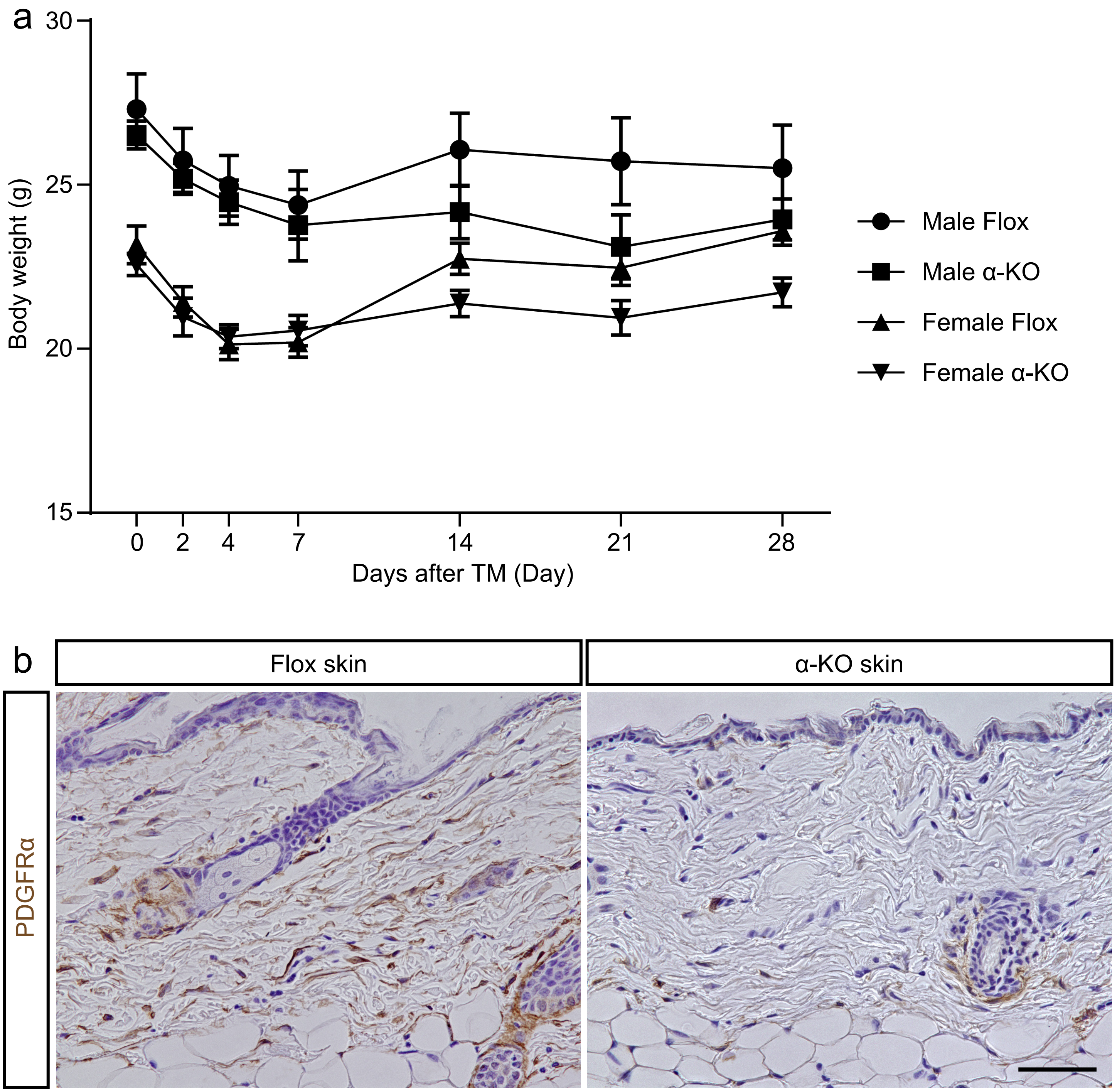
a
Body weight (g)
30
25
20
15
0
2
4
7
14
21
28
Days after TM (Day)
Male Flox
Male α-KO
Female Flox
Female α-KO
b
Flox skin
α-KO skin
PDGFRα

Supplementary Figure 1 | Sponge implantation model in PDGFRα-inactivated (α-KO) mice. (a) The change in the body weights of male Flox mice (n = 12), male α-KO mice (n = 11), female Flox mice (n = 11), and female α-KO mice (n = 11) throughout experiments. (b) Immunohistochemistry of PDGFRα (brown) in Flox mice skin as a positive control (left), and α-KO mice (right) at 14 days after tamoxifen treatment for 5 consecutive days. Sections were counterstained with hematoxylin (pale blue). Scale bar indicates 50 μm.

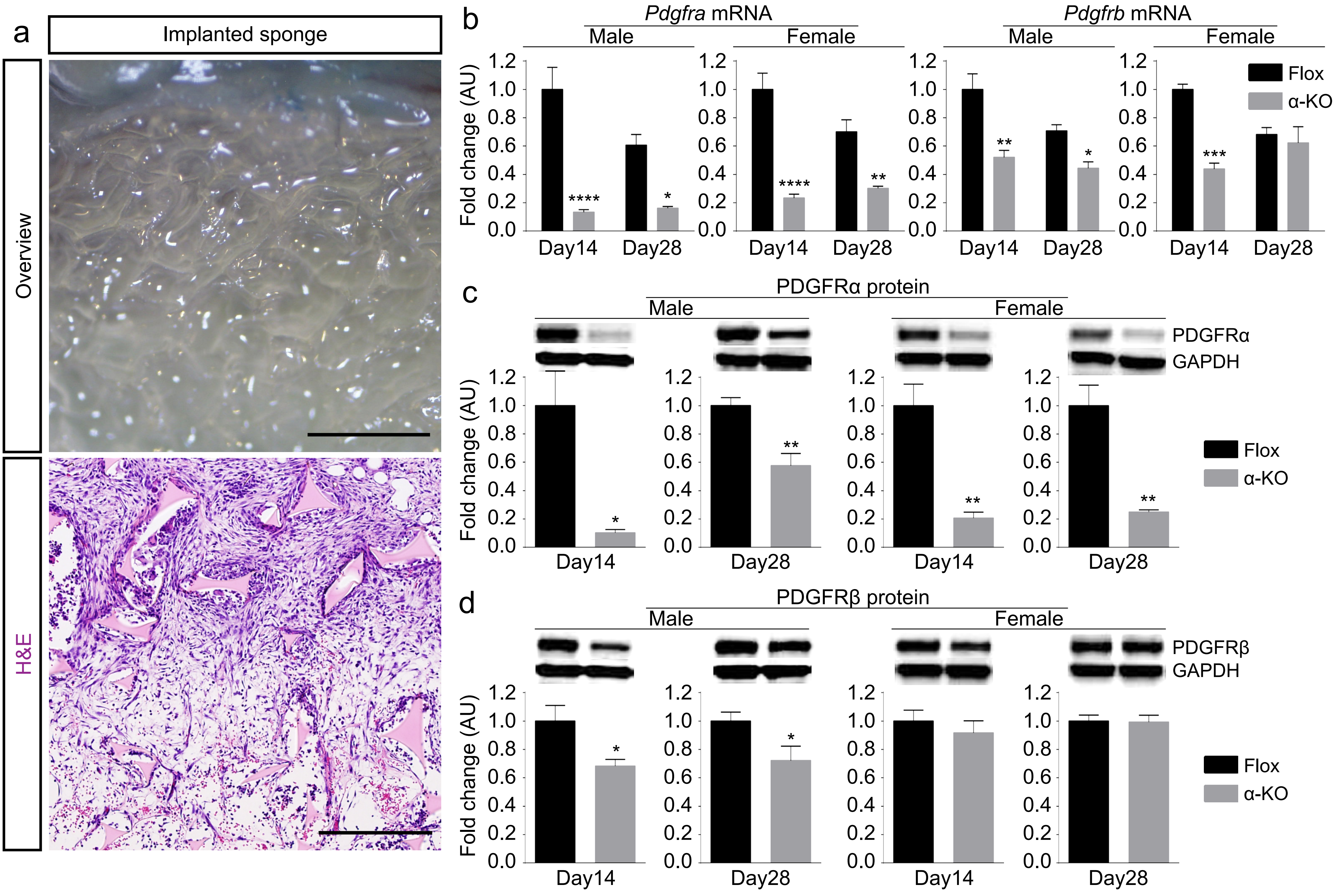

a
Implanted sponge
Overview
H&E
b
Pdgfra mRNA
Pdgfrb mRNA
Male
Female
Fold change (AU)
Day14
Day28
Flox
α-KO
c
PDGFRα protein
PDGFRα
GAPDH
d
PDGFRβ protein
PDGFRβ

Supplementary Figure 2 | Characteristics of the systemic α-KO mice with sponge implantation. (a) Overview of the cut surface (upper) and H&E stained section (bottom) of the implanted sponge into Flox mice, excised after 14 days of implantation. The frame work of the sponge, corresponding to the translucent and angulated objects, was surrounded by connective tissues that grew into sponge. Connective tissue shows different types of cells, including fibroblasts, endothelial cells, and macrophages. Scale bars indicate 1 mm (a, upper) and 300 μm (a, bottom). (b) Real-time PCR analyses of *Pdgfra* and *Pdgfrb* mRNA expression levels in Flox and α-KO mice of both sexes in implanted sponges; n = 4-5 per group. (c, d) Western blot analysis of PDGFRα (c) and PDGFRβ (d) protein expression levels in Flox and α-KO mice of both sexes in implanted sponges; n = 4-5 per group. *, $p < 0.05$ versus Flox mice; **, $p < 0.01$ versus Flox mice; ***, $p < 0.001$ versus Flox mice; ****, $p < 0.0001$ versus Flox mice.

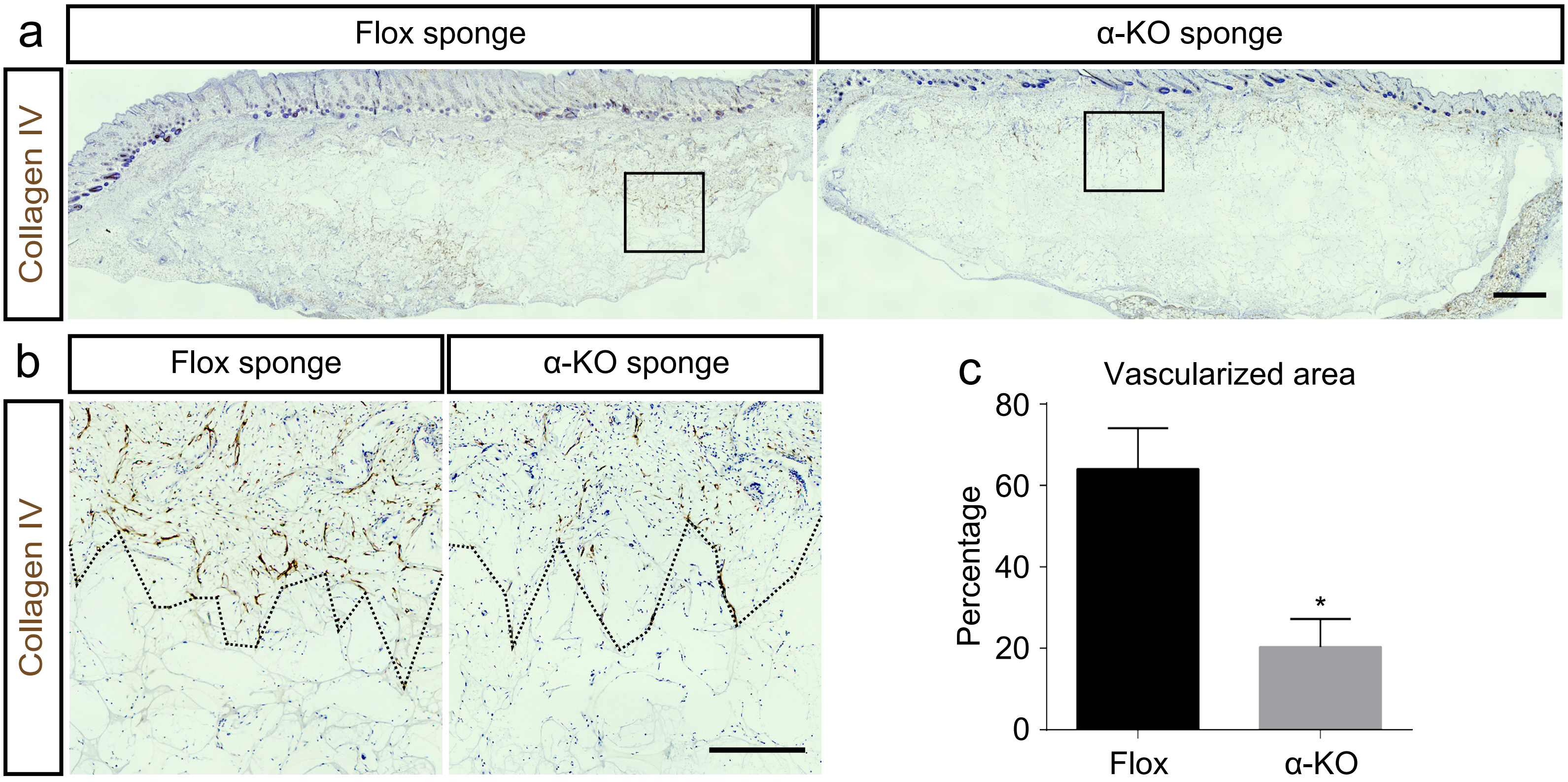
a
Flox sponge
α-KO sponge
Collagen IV
b
Flox sponge
α-KO sponge
Collagen IV
c
Vascularized area
Percentage
80
60
40
20
0
*
Flox
α-KO

Supplementary Figure 3 | Angiogenesis is suppressed in implanted sponges in α-KO mice. (a, b) Immunohistochemical analysis of blood vessel formation in implanted sponge. Immunohistochemistry of collagen type IV (brown), a blood vessel specific extracellular matrix, in cross-sectional area of implanted sponges at day 14 after implantation. Boxed areas in (a) show as high magnification panels in (b) with the vascularized areas delineated by dotted lines. (c) Statistical analysis of the vascularized areas. Using Flox mice as the control (a, left), collagen type IV positive vascularized areas in α-KO (a, right) were examined in male at day 14 after implantation; $n = 4$ mice per group. *, $p < 0.05$ versus Flox mice. Scale bars indicate 1 mm (a) and 500 μm (b), respectively.

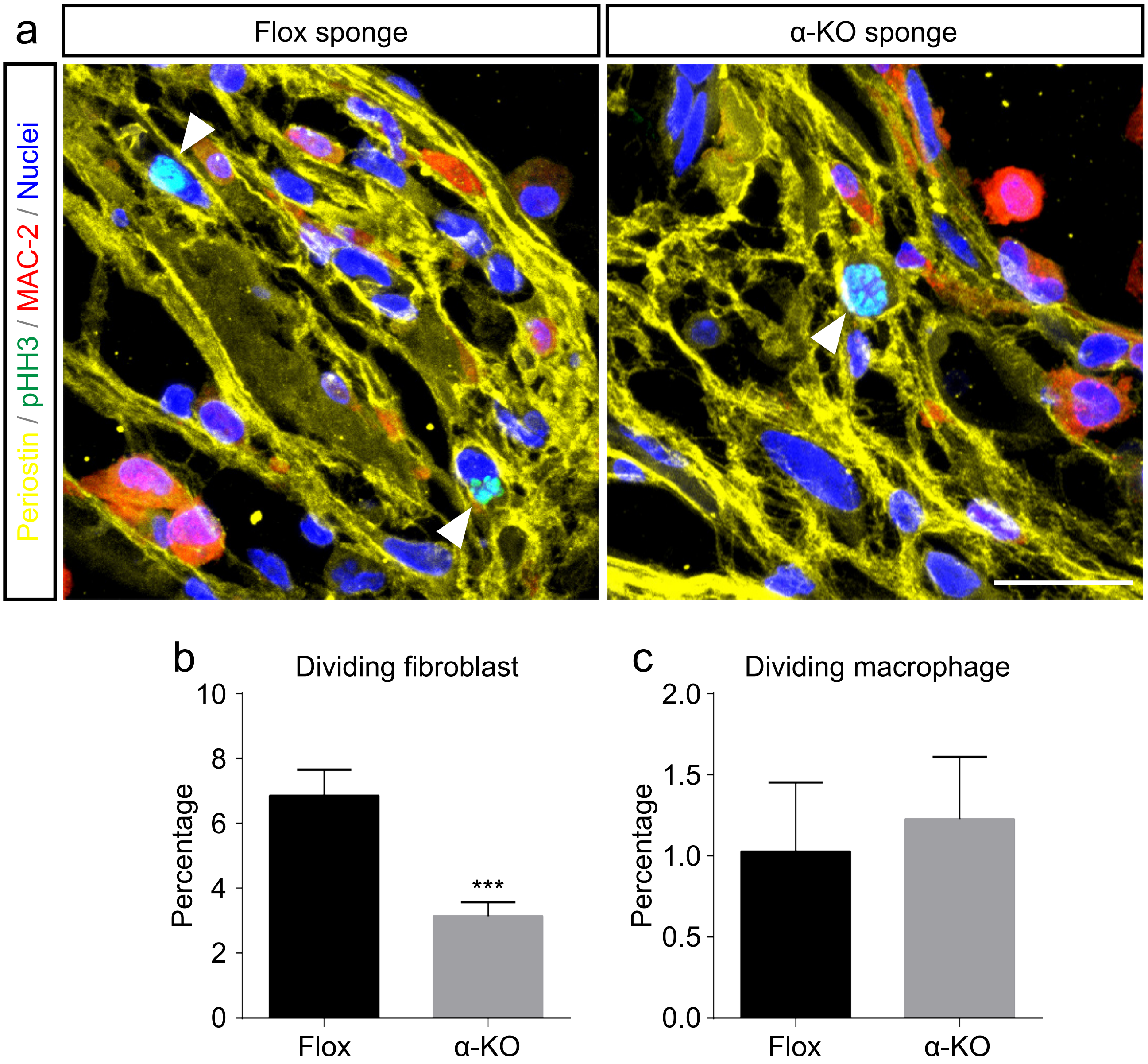
a
Flox sponge
α-KO sponge
Periostin / pHH3 / MAC-2 / Nuclei
b
Dividing fibroblast
Percentage
10
8
6
4
2
0
***
Flox
α-KO
c
Dividing macrophage
Percentage
2.0
1.5
1.0
0.5
0.0
Flox
α-KO

Supplementary Figure 4 | Cell division marker positive fibroblasts are decreased in implanted sponges in α-KO mice. (a) Immunofluorescence of phospho-histone H3 (pHH3), a M phase specific marker, in implanted sponges of Flox and α-KO mice. Number of dividing periostin-positive fibroblasts (yellow) that were co-labeled with pHH3 (green) were decreased in α-KO mice than in Flox mice (arrowheads). Infiltrating macrophages (red) were similar between the two genotypes. Nuclei are depicted by DAPI staining (blue). Scale bar indicates 20 μm. (b, c) Morphometrical analysis of M phase (dividing) cells. The percentage of dividing fibroblasts (pHH3/periostin-positive) in periostin-positive fibroblasts is significantly less in α-KO mice than in Flox mice (b), but that of dividing macrophages (pHH3/MAC-2-positive) in MAC-2-positive macrophage is comparable between the two genotypes (c); $n = 4$ mice per group. ***, $p < 0.001$ versus Flox.

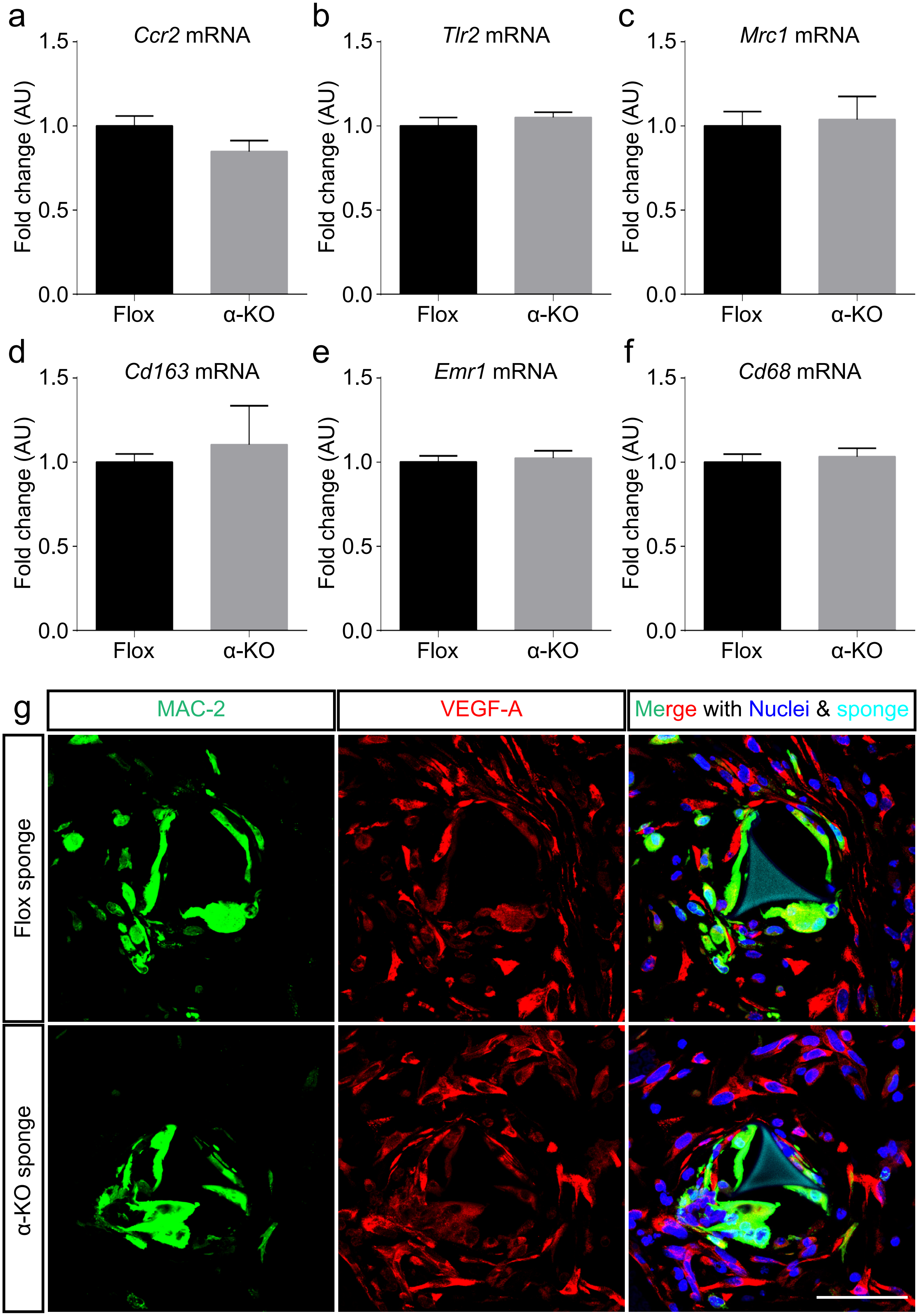
a
Ccr2 mRNA
b
Tlr2 mRNA
c
Mrc1 mRNA
d
Cd163 mRNA
e
Emr1 mRNA
f
Cd68 mRNA
Fold change (AU)
1.5
1.0
0.5
0.0
Flox
α-KO
g
MAC-2
VEGF-A
Merge with Nuclei & sponge
Flox sponge
α-KO sponge

Supplementary Figure 5 | Infiltrating macrophages are comparable between two genotypes. (a-f) Real-time PCR analyses of M1, M2 and pan-macrophage markers in implanted sponges; n = 4 per group. No significant difference can be observed in M1 (*Ccr2* and *Tlr2*; a, b), M2 (*Mrc1* and *Cd163*; c, d) and pan-macrophage markers (*Emr1* and *Cd68*; e, f). (g) VEGF-A (red), a potent angiogenic factor, expresses in MAC-2 positive infiltrated macrophages (green). Nuclei are depicted by DAPI staining (blue), and sponges are visible due to the autofluorescence (cyan). No significant difference in expression levels of VEGF-A can be seen between two genotypes. Scale bar indicates 50 μm.

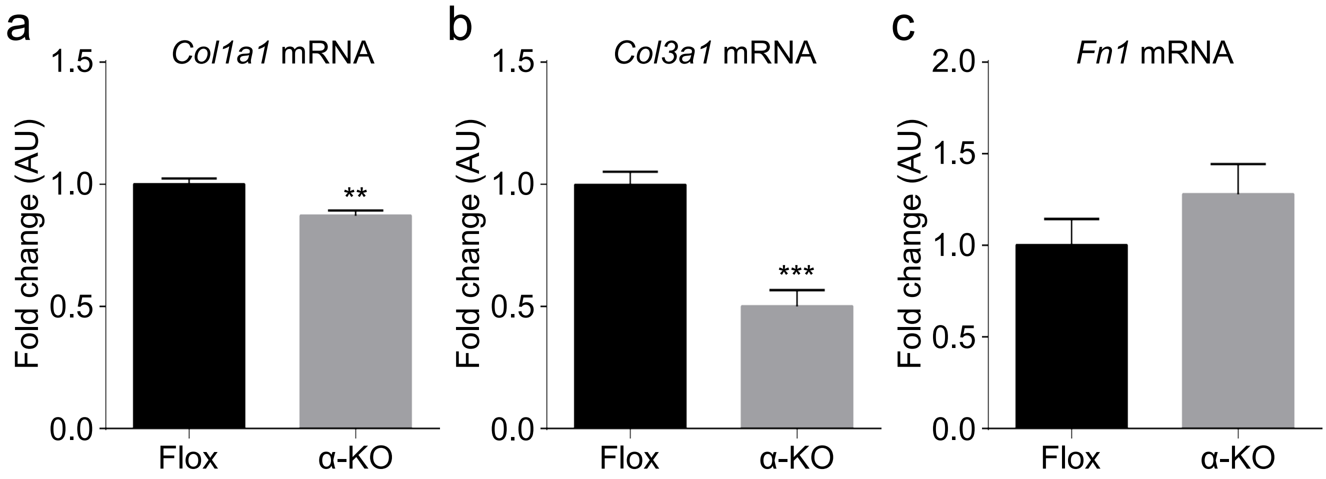

a
Col1a1 mRNA
1.5
1.0
0.5
0.0
Fold change (AU)
**
Flox
α-KO
b
Col3a1 mRNA
1.5
1.0
0.5
0.0
Fold change (AU)
***
Flox
α-KO
c
Fn1 mRNA
2.0
1.5
1.0
0.5
0.0
Fold change (AU)
Flox
α-KO

Supplementary Figure 6 | Collagen expression is significantly suppressed in α-KO fibroblasts. (a-c) Real-time PCR analyses of *Col1a1*, *Col3a1* and *Fn1* mRNA expression levels in Flox and α-KO fibroblasts; n = 6 per group. Expression levels of *Col1a1* and *Col3a1* mRNAs are significantly suppressed in α-KO fibroblasts. In contrast, expression level of *Fn1* mRNA is comparable between two genotypes (n.s.). **, $p < 0.01$ versus Flox fibroblasts; ***, $p < 0.001$ versus Flox fibroblasts.

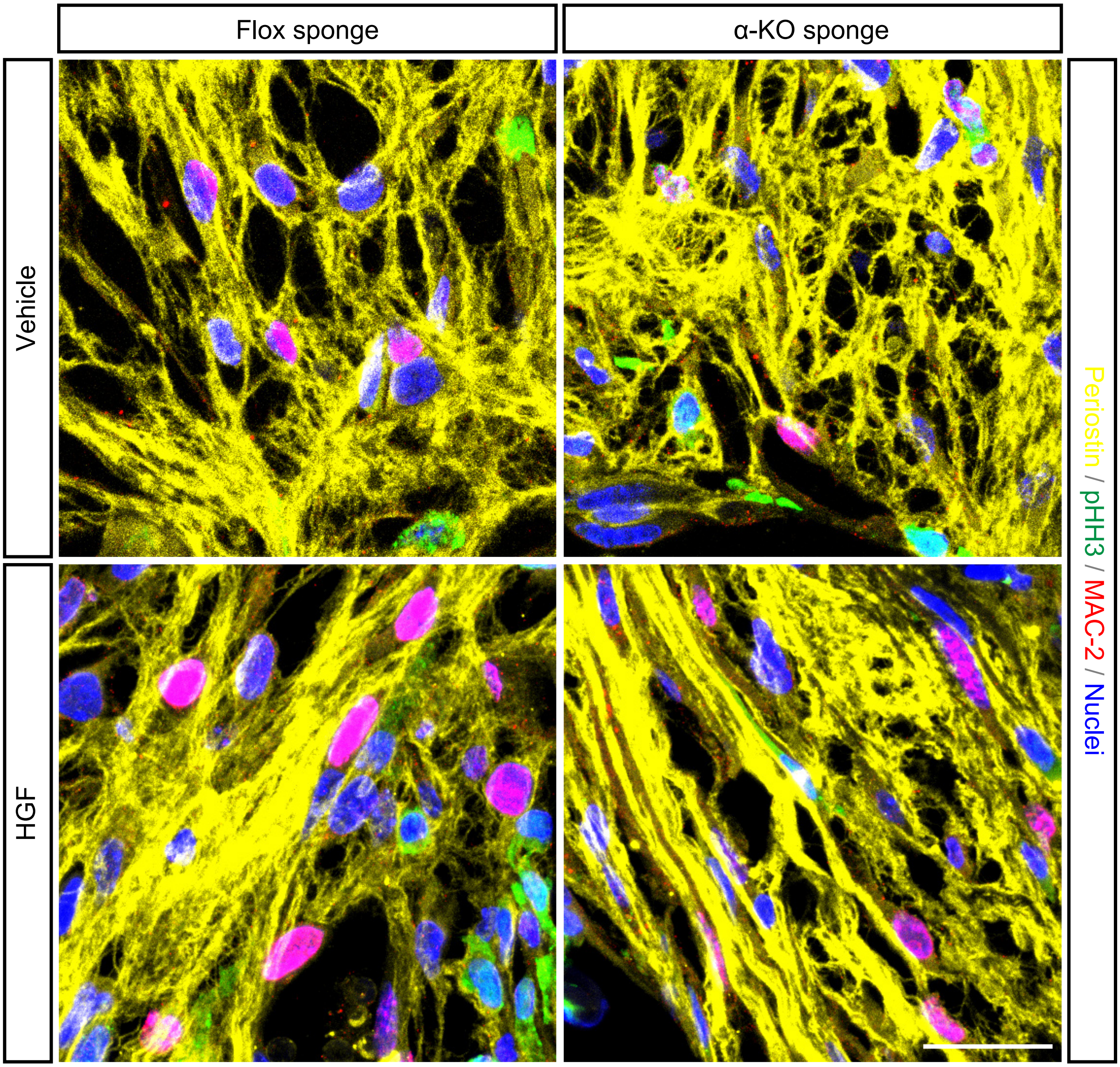
Flox sponge
α-KO sponge
Vehicle
HGF
Periostin / pHH3 / MAC-2 / Nuclei

Supplementary Figure 7 | HGF promotes fibroblast proliferation in implanted sponges in α-KO mice. Immunofluorescence of ingrowing granulation tissue within the implanted sponges of Flox and α-KO mice, with or without HGF treatment. Periostin-positive fibroblasts (yellow) that are co-labeled with Ki67 (red) are increased in HGF treated groups. MAC-2 (green)-positive cells represent infiltrating macrophages into sponges. Increased proliferation of fibroblasts after HGF administration may suggest HGF plays a key role in fibroblast number in sponges. Nuclei are depicted by DAPI staining (blue). Scale bar indicates 20 μm.

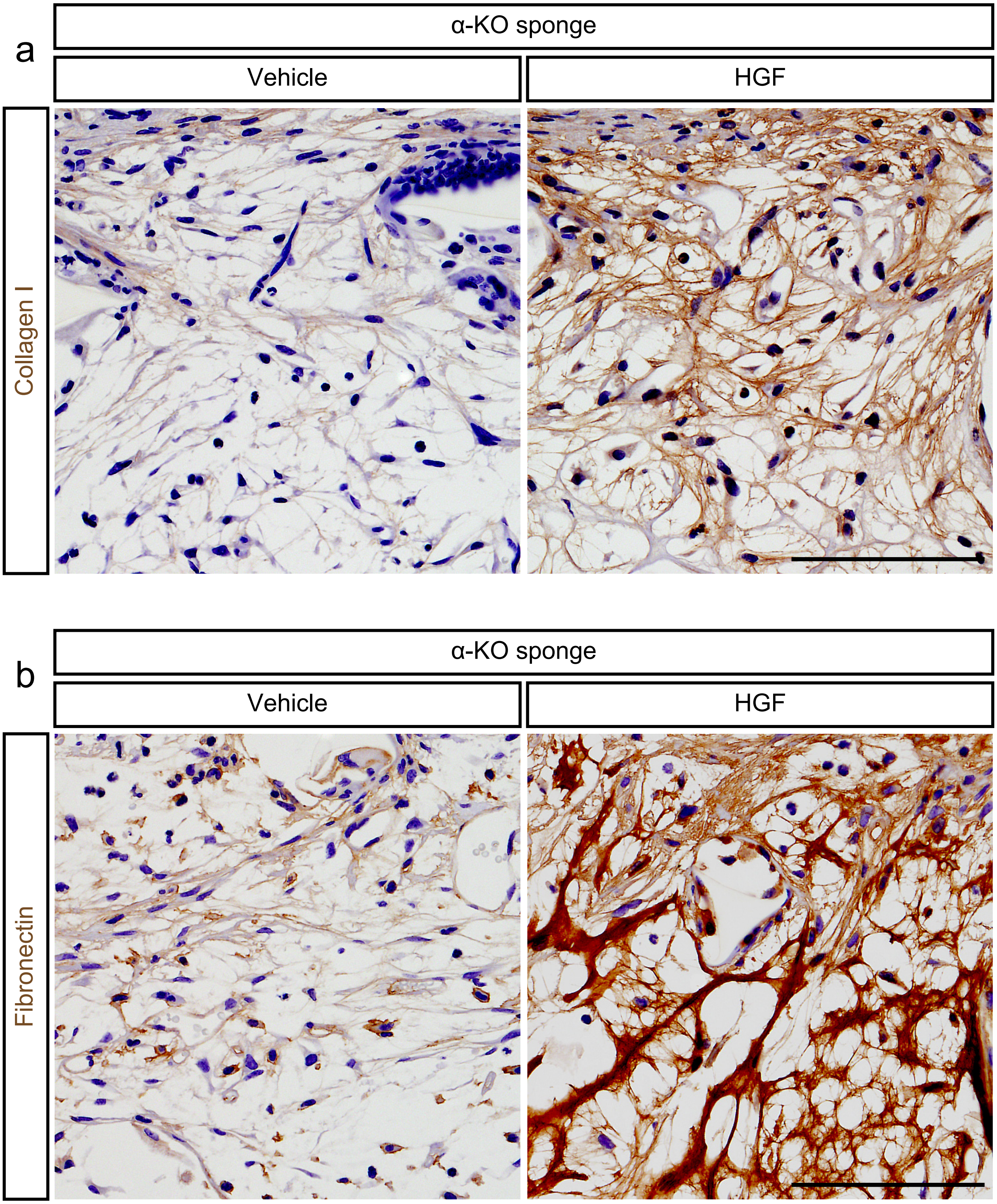
a
α-KO sponge
Vehicle
HGF
Collagen I
b
α-KO sponge
Vehicle
HGF
Fibronectin

Supplementary Figure 8 | HGF promotes ECM deposition in implanted sponges in α-KO mice. (a) Immunohistochemistry of collagen type I in ingrowing granulation tissue within the sponges of α-KO mice with or without HGF treatment. HGF administration into sponges promotes collagen type I deposition. (b) Immunohistochemistry of fibronectin in ingrowing granulation tissue within the sponges of α-KO mice with or without HGF treatment. HGF administration into sponges promotes fibronectin deposition. Scale bar indicates 100 μm.
